# Lis1 Acts as a “Clutch” between the ATPase and Microtubule-Binding Domains of the Dynein Motor

**DOI:** 10.1016/j.cell.2012.07.022

**Published:** 2012-08-31

**Authors:** Julie Huang, Anthony J. Roberts, Andres E. Leschziner, Samara L. Reck-Peterson

**Affiliations:** 1 Department of Cell Biology, Harvard Medical School, Boston, MA 02115, USA; 2 Astbury Centre for Structural Molecular Biology, School of Molecular and Cellular Biology, Faculty of Biological Sciences, University of Leeds, Leeds LS2 9JT, UK; 3 Department of Molecular and Cellular Biology, Harvard University, Cambridge, MA 02138, USA

## Abstract

The lissencephaly protein Lis1 has been reported to regulate the mechanical behavior of cytoplasmic dynein, the primary minus-end-directed microtubule motor. However, the regulatory mechanism remains poorly understood. Here, we address this issue using purified proteins from *Saccharomyces cerevisiae* and a combination of techniques, including single-molecule imaging and single-particle electron microscopy. We show that rather than binding to the main ATPase site within dynein's AAA+ ring or its microtubule-binding stalk directly, Lis1 engages the interface between these elements. Lis1 causes individual dynein motors to remain attached to microtubules for extended periods, even during cycles of ATP hydrolysis that would canonically induce detachment. Thus, Lis1 operates like a “clutch” that prevents dynein's ATPase domain from transmitting a detachment signal to its track-binding domain. We discuss how these findings provide a conserved mechanism for dynein functions in living cells that require prolonged microtubule attachments.

## Graphical abstract

**Graphical Abstract:**

**Figure:**
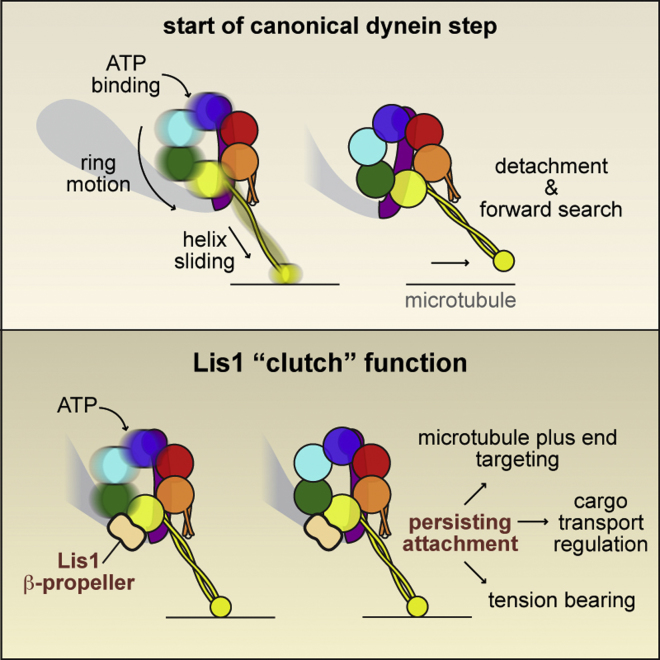

**Highlights:**

► Single-molecule and structural data reveal how Lis1 regulates dynein motility ► Lis1 binds between dynein's ATPase and microtubule-binding domains ► Lis1 alters communication between these elements, prolonging microtubule attachment ► Mutating an arginine finger motif in dynein mimics aspects of Lis1's effect

## Teaser

Lis1 improves dynein processivity by allowing dynein to remain bound to microtubules while it hydrolyzes ATP, a reaction that generally serves to cause motor dissociation.

## Introduction

**Figure 1:**
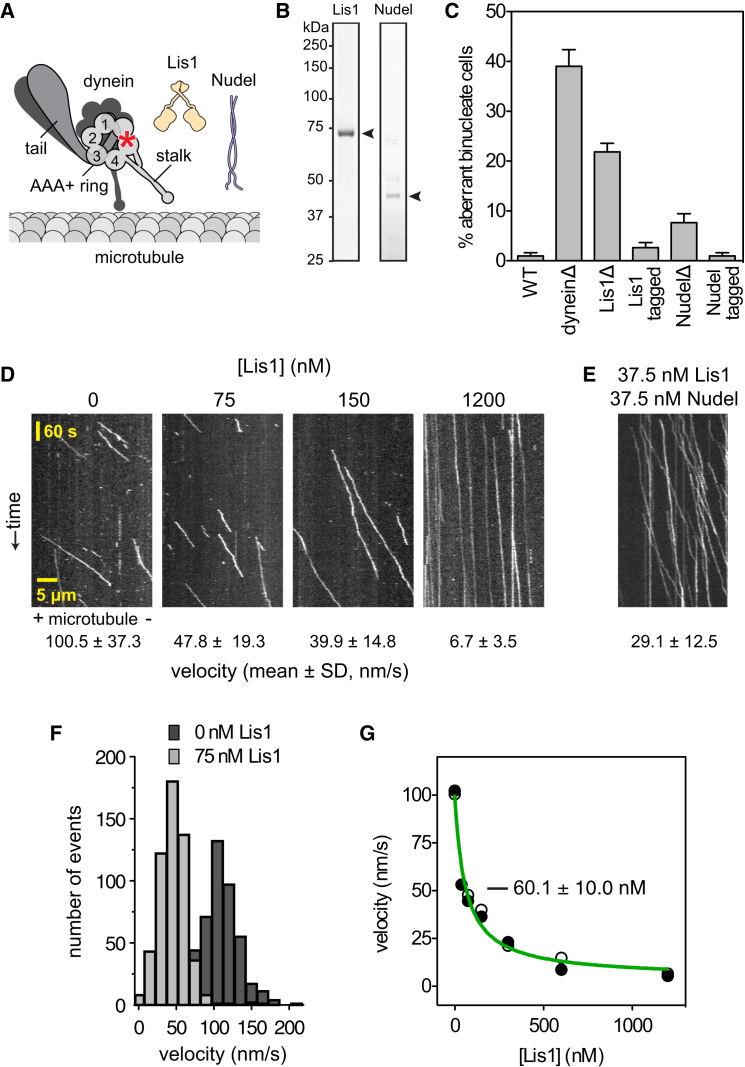
Impact of Lis1 on Single Dynein Molecule Motility (A) Diagram of dynein, Lis1, and Nudel constructs. The C terminus of dynein's heavy chain is tagged with a HaloTag and covalently bound to tetramethylrhodamine (TMR; red asterisk). (B) Purification of yeast Lis1 and Nudel. Lis1 and Nudel were isolated from *S. cerevisiae* using affinity purification tags followed by removal of tags by TEV protease cleavage. SDS-PAGE shows Lis1 and Nudel after the final purification step. Arrowheads indicate Lis1 and Nudel protein. (C) C-terminal SNAP tags on Lis1 and Nudel do not affect their nuclear segregation function in vivo. Cells expressing Lis1 or Nudel modified with the SNAP tag (tagged) have near-WT levels of aberrant binucleate cells. The mean and SE of proportion are shown (n > 206 per data point). (D) Kymographs of TMR-labeled dynein molecules moving along microtubules over time in the presence of increasing concentrations of Lis1. Plus (+) and minus (−) indicate microtubule polarity. The mean velocity for each condition is shown below (n > 100 per data point). For average durations of microtubule association and average run lengths as a function of Lis1 concentration, see Figures S1A–S1D. The effect of Lis1 on kinesin motility is shown in Figures S1E and S1F. (E) Kymograph of TMR-labeled dynein molecules in the presence of 37.5 nM Lis1 and 37.5 nM Nudel, with mean velocity below (n = 217). Time and distance scales are as in (D). Additional quantification is shown in Figures S1G and S1H. (F) Histogram showing the velocity distribution of single dynein molecules in the absence of Lis1 (dark gray; n = 498) and at one example Lis1 concentration (75 nM; light gray; n = 539). At all Lis1 concentrations tested (37.5, 75, 150, 300, 600, and 1,200 nM), the dynein velocity distribution was unimodal and could be well fit by a single Gaussian (*R*^2^ values between 0.8448 and 0.9894; data not shown). (G) Plot of dynein velocity as a function of Lis1 concentration. Data points from two independent dynein preparations are shown (black and white circles; n > 100 per data point). Data were fit to the following equation: *V* = *V*_0_ (1 − [(*F*_max_[Lis1]) / (*K*_1/2_ + [Lis1])]), in which *V* = velocity at a given Lis1 concentration, [Lis1]; *V*_0_ = velocity of dynein in the absence of Lis1; *F*_max_ = maximal fractional velocity reduction; and *K*_1/2_ = [Lis1] required for half maximal velocity reduction. The best-fit values (±error of the fit) are *V*_0_ = 100.0 ± 3.506 nm/s, *F*_max_ = 0.957 ± 0.031, and *K*_1/2_ = 60.1 ± 10.0 nM. The *R*^2^ value is 0.9797. See also Table S2A.

To help create the complex internal organization within eukaryotic cells, the myosin and kinesin motor protein families have undergone widespread gene duplication and evolution, presumably so they can fulfill particular functional niches (e.g., transporting cargos, bearing tension, and sliding filaments; Vale, 2003). Accordingly, their members show a spectrum of properties, with different catalytic rates and structural adaptations. A striking departure from this pattern is one of the least understood cytoskeletal motors, cytoplasmic dynein (Figure 1A). This one motor powers nearly all movement toward the microtubule minus end in most eukaryotic cells. Its many functions include transporting and positioning diverse cargos (e.g., mRNAs, proteins, and organelles) during interphase, exerting tension between the microtubule network and cell cortex during cell migration, and helping to construct the spindle during mitosis and meiosis (Karki and Holzbaur, 1999; Vale, 2003). Regulation of dynein is therefore critical, and human diseases arise from its dysfunction (Gerdes and Katsanis, 2005). In addition to task-specific regulators, cytoplasmic dynein has three ubiquitous cofactors that are required for most, if not all, of its functions across eukaryotes (Kardon and Vale, 2009; Vallee et al., 2012): the dynactin complex and two proteins analyzed in this study, Lis1 and Nudel.

Dynein's ATP-hydrolyzing and track-binding elements are key potential targets for regulation and differ in three significant ways from those of myosin and kinesin. First, rather than a compact fold, dynein's ATPase domain is ring shaped, containing six covalently linked AAA+ modules (Neuwald et al., 1999; Roberts et al., 2009). Second, rather than a single site of ATP binding/hydrolysis, four of dynein's AAA+ modules (AAA1–AAA4) can bind nucleotide, with AAA1 being the main site of ATP hydrolysis (Cho et al., 2008; Gibbons et al., 1991; Kon et al., 2004, 2012; Schmidt et al., 2012). Finally, instead of close integration of the track-binding and hydrolysis sites, these domains in dynein are spatially separated by a ∼10 nm intramolecular coiled-coil stalk, which protrudes from AAA4 (Cho and Vale, 2012).

Together, dynein's architectural features make interdomain communication a central part of its mechanism and an additional potential target for regulation. For example, structural changes within dynein's AAA+ ring, driven by ATP binding and hydrolysis at AAA1, produce cyclic changes in the affinity of the microtubule-binding domain at the stalk's tip (Imamula et al., 2007; Kon et al., 2004, 2009). In some distantly related AAA+ ATPases, pulses of structural changes are transmitted around the ATPase ring with the assistance of arginine finger motifs that reach from one subunit into the active site of the neighboring subunit (Erzberger and Berger, 2006). However, the extent to which dynein uses similar mechanisms is unknown.

A strong candidate for altering the structural changes within dynein is the evolutionarily conserved protein Lis1. Mutations in the human *LIS1* gene cause classical lissencephaly, a severe brain development disorder (Reiner et al., 1993). In brain slices and cultured cells, perturbing Lis1 levels causes defects in dynein-mediated processes such as nuclear migration, mitosis, and cargo transport (Ding et al., 2009; Faulkner et al., 2000; Pandey and Smith, 2011; Smith et al., 2000; Tai et al., 2002; Tanaka et al., 2004; Tsai et al., 2007; Yi et al., 2011). Like cytoplasmic dynein's force-generating subunit, Lis1 is dimeric. Each Lis1 protomer contains an N-terminal dimerization domain and a C-terminal β-propeller domain that binds to the dynein motor domain (Figure 1A; Sasaki et al., 2000; Tai et al., 2002; Tarricone et al., 2004). A coiled-coil protein, Nudel (or its paralogue, NudE), is thought to further tether Lis1 to dynein (McKenney et al., 2010), allowing Lis1 to function at lower concentrations in vivo (Efimov, 2003; Li et al., 2005; Wang and Zheng, 2011; Żyłkiewicz et al., 2011). In *Saccharomyces cerevisiae*, the Lis1 and Nudel orthologs (Pac1 and Ndl1, respectively) serve to concentrate dynein at the plus ends of microtubules (Lee et al., 2003; Li et al., 2005; Sheeman et al., 2003). This is a critical step in targeting dynein to the cell membrane, where it positions the nucleus during cell division.

A range of properties has been ascribed to Lis1, including slowing dynein-driven microtubule sliding in multiple motor assays, prolonging dynein stall events under load, and enhancing the microtubule affinity of dynein when ADP and V_i_ (a phosphate analog) are bound (McKenney et al., 2010; Torisawa et al., 2011; Yamada et al., 2008). However, Lis1's mechanism of action remains largely mysterious, in part because of the lack of a recombinant system in which both Lis1 and dynein can be manipulated and studied at the single-molecule level. Although reports have suggested that Lis1 binds the AAA1 module of dynein (Sasaki et al., 2000; Tai et al., 2002), this has not been tested with functional proteins, and structural information on the dynein/Lis1 complex is not yet available. Moreover, reconciling the reported in vitro effects of Lis1 and Nudel with specific dynein activities in vivo has been controversial (Allan, 2011).

Here, using functional, recombinant proteins from *S. cerevisiae*, we show that Lis1 engages dynein at AAA3/AAA4. From this site, Lis1 acts like a “clutch” to regulate communication between dynein's catalytic ring and microtubule-binding stalk, promoting a microtubule-bound state. Supporting these results, we identify mutations at the AAA3/4 junction that drastically impair Lis1 binding and motility regulation in vitro and dynein function in vivo. In addition, we identify an arginine finger motif within AAA4 and find that its mutation mimics aspects of Lis1's effect in vitro. Previous genetic studies in an evolutionarily distant filamentous fungus showed that the same mutation can partially rescue Lis1 loss in vivo (Zhuang et al., 2007). These results allow us to propose how Lis1 biases dynein to a microtubule attached-state and assists in a variety of cellular functions across eukaryotes.

## Results

### Lis1 Promotes a Microtubule-Attached State in Single Dynein Molecules

To dissect how Lis1 and Nudel regulate dynein's motility, we wanted a system in which all three components could be manipulated and studied at the single-molecule level (Figure 1A). We previously developed methods to purify native cytoplasmic dynein complexes from *S. cerevisiae*, fluorescently label them with tetramethylrhodamine (TMR; via a C-terminal HaloTag), and visualize their movement along immobilized microtubules (Reck-Peterson et al., 2006). Here, we purified Lis1 and Nudel (Figure 1B), which were expressed from their genomic loci in *S. cerevisiae*. We inserted cleavable tags for affinity purification and a C-terminal SNAP-tag (giving the option of covalent labeling). The 20-kDa SNAP-tag did not interfere with the function of either protein, as revealed in nuclear segregation assays (Figure 1C). We eliminated the risk of endogenous regulators (Lis1, Nudel, and dynactin) from copurifying with dynein via genomic deletions (Table S1 available online).

**Figure S1:**
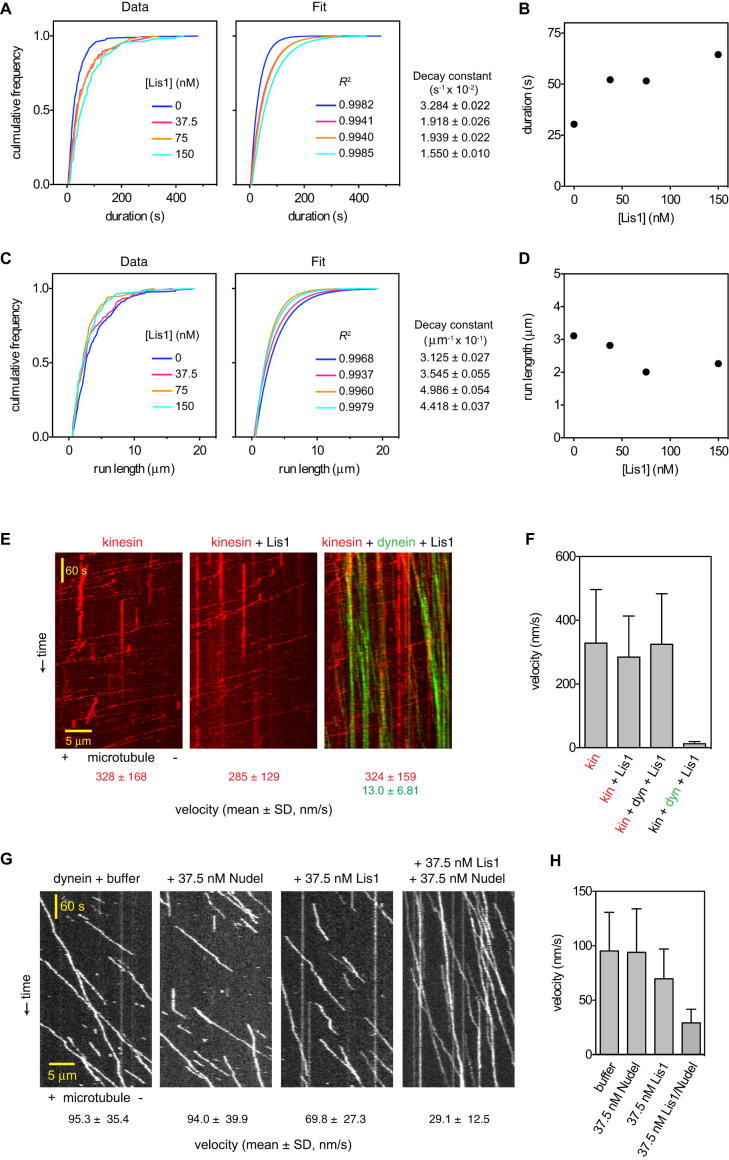
Impact of Lis1 and Nudel on Kinesin and Dynein Motility, Related to Figure 1 (A) Cumulative frequency plots of the duration of dynein's microtubule association at varying Lis1 concentrations (left) and their associated fits to a one-phase exponential decay (right). *R*^2^ values and decay constants (± error of the fit) are shown at right and far right, respectively. At Lis1 concentrations exceeding 150 nM, dynein molecules frequently remained attached beyond the last frame of the movie and were thus not included in the analysis. Data were pooled from two independent experiments (n = 125–251 per data point). (B) Plot of the average duration of dynein's microtubule association as a function of Lis1 concentration. SE of the fit for each data point ranges from 0.201 to 0.711 s. (C) Cumulative frequency plots of dynein run length at varying Lis1 concentrations (left) and their associated fits to a one-phase exponential decay (right). *R*^2^ values and decay constants (± error of the fit) are shown at right and far right, respectively. Data were pooled from two independent experiments (n = 118–243 per data point). (D) Plot of average run lengths of dynein as a function of Lis1 concentration. SE of the fit for each point ranges from 0.019 to 0.044 μm. Dynein run length does not show a strong dependency on Lis1 concentration, due to two combined effects of Lis1 on dynein motility: although dynein remains attached to the microtubule for longer, its velocity is slower, so there is little change in the average travel distance. (E) Kymographs of GFP-tagged kinesin (red) molecules moving along microtubules over time, either alone or in the presence of 600 nM Lis1 and TMR-labeled dynein (green). Plus (+) and minus (−) indicate microtubule polarity. The mean velocity for each motor is shown below. The human kinesin 1–560 aa (K560) construct was expressed and purified from *Escherichia coli* as previously described (Case et al., 1997). (F) Mean single molecule velocities from (E). Error bars show SD. The velocity shown corresponds to highlighted motor (n > 141 per data point). (G) Kymographs of TMR-labeled dynein molecules moving on microtubules in the presence and absence of 37.5 nM Lis1 and/or 37.5 nM Nudel. The mean velocity for each condition is shown below. Plus (+) and minus (−) indicate microtubule polarity. (H) Quantification of single dynein molecule velocities (mean ± SD) from (G). Nudel enhances the Lis1-dependent reduction of dynein velocity (n > 216 per data point).

We began by observing dynein motility at the single-molecule level over a range of Lis1 concentrations. In the absence of Lis1, dynein bound to and traveled along microtubules with a mean velocity of ∼100 nm/s (Figure 1D), similar to previous reports (Reck-Peterson et al., 2006). As the Lis1 concentration increased, two trends were apparent. First, dynein velocity became progressively slower, reaching ∼7 nm/s at the highest Lis1 concentration tested (1,200 nM). This can be appreciated in the kymographs in Figure 1D, in which steeper lines indicate slower movement. Second, Lis1 increased the length of time that dynein remained attached to the microtubule. For example, at 1,200 nM Lis1, many dynein molecules were attached for the entire duration of a 10 min movie (seen in Figure 1D as lines that span the kymograph's y axis; see Figures S1A–S1D for quantification). Control assays with kinesin showed that the effect of Lis1 is specific to dynein (Figures S1E and S1F). Thus we conclude that Lis1 can convert dynein to a mechanochemical state in which its velocity is slowed and its microtubule attachments are prolonged.

Notably, at a given Lis1 concentration, the population of dynein molecules responded similarly: velocity histograms remained unimodal as the mean decreased (Figure 1F). We plotted dynein's velocity as a function of Lis1 concentration. The plot is well fitted by a hyperbolic curve with a maximal velocity reduction of 95.7 ± 3.1% (Figure 1G; Table S2A). These data show that saturating concentrations of Lis1 can almost entirely arrest dynein on the microtubule. The Lis1 concentration giving half-maximal velocity reduction (*K*_1/2_) in this assay is 60.1 ± 10.0 nM. Hereafter, we refer to dynein in the presence of near-saturating Lis1 concentrations (>5-fold higher than the *K*_1/2_) as dynein/Lis1.

We next tested whether Nudel, Lis1 and dynein's binding partner, affects the behavior of single dynein molecules in vitro. At 37.5 nM, Nudel alone had little effect on dynein motility (Figures S1G and S1H). By contrast, a mixture of 37.5 nM Nudel and 37.5 nM Lis1 elicited a change in dynein behavior akin to that induced by a ∼5-fold higher Lis1 concentration. The mean velocity was slowed to ∼29 nm/s, and microtubule encounters were markedly prolonged (Figures 1E and S1H). These results show interesting similarities and differences compared with earlier work. In previous in vitro experiments, mammalian Nudel was found to dissociate dynein from microtubules and suppress Lis1's effect on the velocity of unloaded dynein (McKenney et al., 2010; Yamada et al., 2008). In contrast, we find that Nudel from *S. cerevisiae* allows Lis1 to exert its effect on dynein at lower concentrations even in unloaded conditions. This matches observations from previous experiments in living fungi and frog egg extracts, in which increasing Lis1's concentration rescued Nudel deletion or depletion, respectively (Efimov, 2003; Li et al., 2005; Wang and Zheng, 2011; Żyłkiewicz et al., 2011).

### The Lis1 β Propeller Domain Contains Regulatory Elements that Act on Dynein's Motor

**Figure 2:**
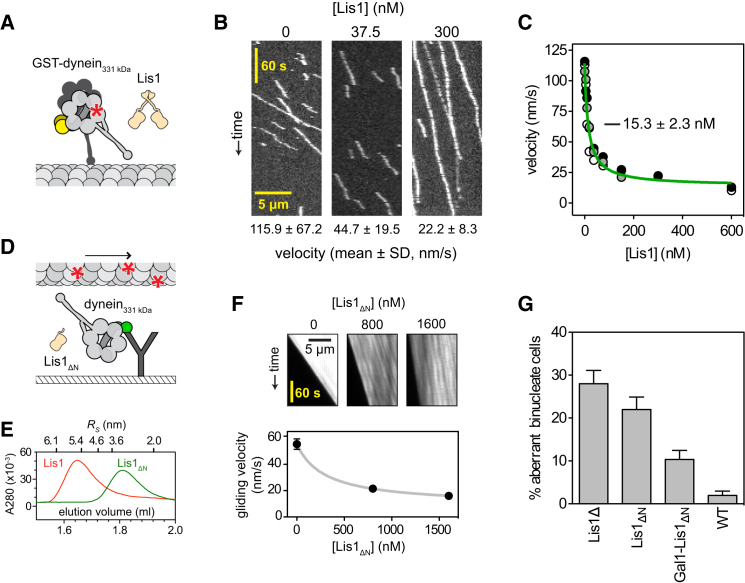
Defining Structural Domains Required for Motility Regulation by Lis1 (A) Diagram of GST-dynein_331 kDa_ and Lis1 constructs. The GST dimerization domain is shown in yellow, and the C terminus is tagged with a HaloTag and covalently bound to TMR (red asterisk). (B) Kymographs of TMR-labeled GST-dynein_331 kDa_ molecules in the presence of increasing concentrations of Lis1. The mean velocity for each condition is shown below (n > 200 per data point). See also Table S2B. For average durations of microtubule association and average run lengths as a function of Lis1 concentration, see also Figures S2A–S2D. (C) Plot of GST-dynein_331 kDa_ velocities as a function of Lis1 concentration. Data points from three independent dynein preparations are shown (black, gray, and white circles; n > 104 per data point). Data were fit as described in Figure 1G (*R*^2^ = 0.9652; *V*_0_ = 114.4 ± 3.531 nm/s, *F*_max_ = 0.877 ± 0.025, and *K*_1/2_ = 15.3 ± 2.3 nM). (D) Diagram of microtubule gliding assay. Monomeric GFP-dynein_331 kDa_ molecules are immobilized to the coverslip via antibodies to GFP (green). Dynein-driven gliding of microtubules (labeled with TMR; red asterisks) is visualized in the presence or absence of monomeric Lis1 (Lis1_ΔN_). (E) Elution profiles of dimeric Lis1 (red) and monomeric Lis1_ΔN_ (green) from a size-exclusion column. Elution volumes of standards with known Stokes radii (*R*_*S*_) are shown above. The calculated Stokes radii of dimeric Lis1 and Lis1_ΔN_ are 5.3 nm and 3.3 nm, respectively. See also Figure S2E. (F) Upper: kymographs of taxol-stabilized microtubules moving via dynein-dependent gliding in the presence of increasing amounts of Lis1_ΔN_. Kymographs show one end of the microtubule. Lower: plot of mean velocities (±SD; n = 10–21) fit as described in Figure 1G (*R*^2^ = 0.9742; *V*_0_ = 55.48 ± 0.6759 nm/s, *F*_max_ = 0.814 ± 0.059, and *K*_1/2_ = 299.1 ± 97.1 nM). The effect of Lis1_ΔN_ on kinesin-dependent microtubule gliding is shown in Figure S2F. (G) Overexpression of Lis1_ΔN_ (Gal1-Lis1_ΔN_) partially rescues the aberrant binucleate phenotype of *S. cerevisiae* cells lacking Lis1 (Lis1Δ). Cells expressing dimeric Lis1 (WT) or monomeric Lis1 (Lis1_ΔN_) under the control of the endogenous Lis1 promoter are as indicated. The mean and SE of proportion are shown (n > 201 per data point). See also Figure S2G.

**Figure S2:**
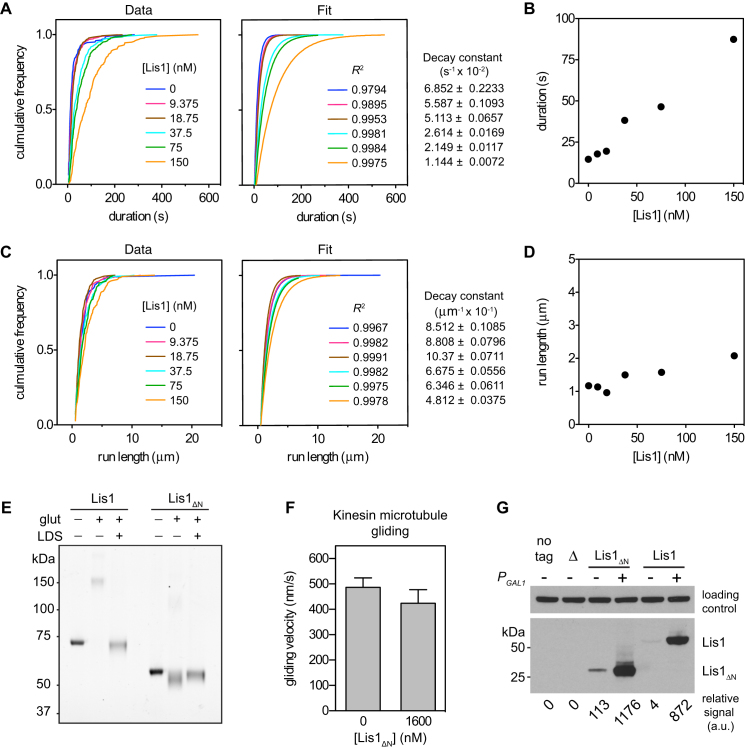
Further Analysis of Dimeric and Monomeric Lis1 Constructs, Related to Figure 2 (A) Cumulative frequency plots of the duration of GST-dynein_331 kDa_ microtubule association in the presence of varying Lis1 concentrations (left) and their associated fits to a one-phase exponential decay (right). *R*^2^ values and decay constants (±error of the fit) are shown at right and far right, respectively. Data were pooled from two independent experiments (n = 220–311 per data point). (B) Plot of the average duration of GST-dynein_331 kDa_ microtubule association as a function of Lis1 concentration. SE of the fit for each data point ranges from 0.247 to 0.554 s. (C) Cumulative frequency plots of GST-dynein_331 kDa_ run length at varying Lis1 concentrations (left) and their associated fits to a one-phase exponential decay (right). *R*^2^ values and decay constants (± error of the fit) are shown at right and far right, respectively. Data were pooled from two independent experiments (n = 206–286 per data point). (D) Plot of average run lengths of GST-dynein_331 kDa_ as a function of Lis1 concentration. SE of the fit for each point ranges from 0.007 to 0.016 μm. As with full-length dynein (Figure S1D), the GST-dynein_331 kDa_ run length does not show a strong dependency on Lis1 concentration. Although dynein remains attached to the microtubule for a longer time, its velocity is slower, so there is little change in the average travel distance. (E) SDS-PAGE of Lis1 and Lis1_ΔN_ after treatment with the crosslinking reagent glutaraldehyde (glut). Exposure of intact Lis1 to glutaraldehyde produces a covalently crosslinked dimer that migrates about twice the size of the untreated form. As a negative control, pretreatment of Lis1 with LDS (4%) to denature Lis1 abolishes formation of the crosslinked dimer. For the Lis1_ΔN_ construct, no shift to a dimeric form is observed in the presence of glutaraldehyde, indicating that it is monomeric. (F) A high concentration (1600 nM) of Lis1_ΔN_ has little effect on the gliding velocity of taxol-stabilized microtubules driven by kinesin. Error bars represent the SD (n = 40 microtubules in each case). (G) Western blots of cellular extracts from galactose-induced cells expressing ZZ-Lis1_ΔN_ or ZZ-Lis1 under the control of either the galactose-inducible promoter (*P*_*GAL1*_; +) or the endogenous Lis1 promoter (−). Cells lacking Lis1 (Δ) or expressing Lis1 lacking the ZZ-tag used for western detection (no tag) were examined in parallel. Protein expression analysis was performed as described (Huang et al., 2006) using a FastPrep-24 (MP Biomedicals). Clarified samples were loaded onto 4%–12% Tris-Bis protein gels (Invitrogen), blotted to nitrocellulose membrane, and probed with a 1:7500 dilution of peroxidase-conjugated anti-ProteinA (PAP) antibody (Sigma). Chemiluminescence signals were detected with autoradiography film and scanned at 800 dpi using a high-resolution film scanner (Aztek Plateau). Relative signals were quantified using ImageJ (National Institutes of Health). The loading control is a nonspecific band that cross-reacts with the PAP antibody.

To test which structural domains are required for Lis1 to regulate dynein motility, we analyzed the motility of a well-characterized dimeric motor domain construct (GST-dynein_331 kDa_; Reck-Peterson et al., 2006). GST-dynein_331 kDa_, which lacks the N-terminal tail, endogenous dimerization motif, and binding sites for associated dynein subunits, contains two motor domains dimerized by GST (Figure 2A) and displays processive motility similar to that of native dynein. In the absence of Lis1, GST-dynein_331 kDa_ traveled along microtubules with a mean velocity of ∼116 nm/s (Figure 2B). Strikingly, Lis1 also reduced the velocity and prolonged microtubule attachments of GST-dynein_331 kDa_ in a dose-dependent manner (Figures 2B and S2A–S2D; Table S2B). The maximal velocity reduction was similar to that observed in native dynein (Figure 2C), but the *K*_1/2_ of Lis1 is lower (∼15 nM). This difference is consistent with the notion that the affinity of Lis1 for dynein increases when dynein's tail domain is removed (Markus et al., 2009; Reck-Peterson et al., 2006). These results indicate that Lis1 regulates dynein motility by acting on elements within the motor domain.

We next tested whether dimerization of either Lis1 or dynein is an essential part of the regulatory mechanism, using purified monomeric dynein_331 kDa_, whose motor activity can be analyzed using a microtubule gliding assay (Figure 2D). To produce monomeric Lis1 (Lis1_ΔN_), we removed Lis1's N-terminal dimerization domain. The monomeric state of Lis1_ΔN_ was verified by size-exclusion chromatography (Figure 2E) and crosslinking (Figure S2E). Monomeric dynein_331 kDa_ drove microtubule gliding at a velocity of 55.5 nm/s (Figure 2F). Significantly, in the presence of 800 nM Lis1_ΔN_, gliding velocity slowed by ∼60% (to 21.5 nm/s) and decreased by ∼70% (to 16.2 nm/s) when the Lis1_ΔN_ concentration was increased to 1,600 nM. These data indicate that a single β-propeller domain of Lis1 can regulate the activity of a single dynein motor domain, provided it is supplied at an elevated concentration (perhaps explaining why similar effects were not observed in a previous study; Torisawa et al., 2011).

We next tested these findings in living cells. Yeast expressing monomeric Lis1_ΔN_ as the sole source of Lis1 exhibited a strong nuclear segregation defect (∼22%; Figure 2G), consistent with malfunction of the dynein pathway. However, when the cellular concentration of Lis1_ΔN_ was increased >10-fold with the use of a strong, galactose-inducible promoter (Figure S2G), the percentage of cells with a nuclear segregation defect was rescued to ∼10% (Figure 2G). This suggests that a monomeric Lis1 construct comprising the β-propeller domain can regulate dynein both in vitro and in vivo, provided its concentration is sufficiently high. Thus, dimerization serves to enhance the apparent affinity between dynein and Lis1.

### Lis1 Alters Allosteric Communication between Dynein's ATPase and Microtubule-Binding Domains

**Figure 3:**
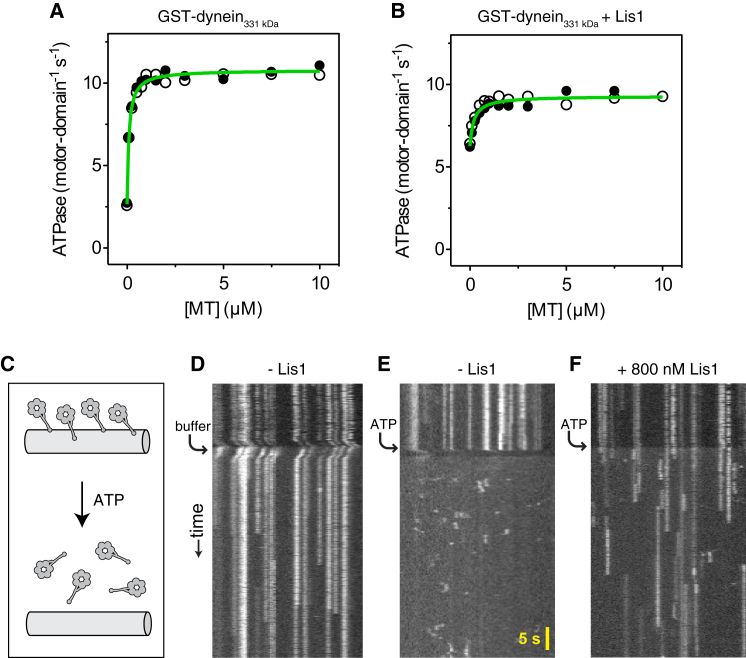
Lis1-Induced Changes in Dynein Mechanochemistry (A) Microtubule-stimulated ATPase activity of GST-dynein_331 kDa_ in the absence of Lis1. Data points from two dynein preparations are shown (black and white circles). Data were fit to the following equation: *k*_obs_ = (*k*_cat_ − *k*_basal_) − [MT]/(*K*_m_(MT) + [MT]) + *k*_basal_. The basal ATPase rate (*k*_basal_) of 2.7 ± 0.2 motor-domain^−1^ s^−1^ (± SE of fit) is activated by microtubules to a maximal rate (*k*_cat_) of 10.8 ± 0.5 motor-domain^−1^ s^−1^. The *K*_m_(MT) is 0.09 ± 0.01 μM (i.e., the microtubule concentration that gives half-maximal activation). (B) Microtubule-stimulated ATPase activity of GST-dynein_331 kDa_ in the presence of 140 nM Lis1. Data points from two dynein preparations are shown (black and white circles). Data were fit as described in (A). The basal ATPase rate of 6.3 ± 0.2 motor-domain^−1^ s^−1^ is activated by microtubules to a maximal rate of 9.3 ± 0.1 motor-domain^−1^ s^−1^. The *K*_m_(MT) is 0.20 ± 0.05 μM. (C) Diagram of the single-molecule microtubule release assay. TMR-labeled monomeric dynein_331 kDa_ molecules are bound to microtubules in the absence of ATP. Perfusion with ATP causes release. (D–F) Kymographs of TMR-labeled dynein_331 kDa_ molecules. After prebinding to microtubules in the absence of ATP, dynein_331 kDa_-TMR molecules are monitored for release and rebinding upon perfusion of (D) buffer lacking ATP, (E) 5 mM ATP, or (F) 5 mM ATP in the presence of 800 nM Lis1. Similar results were observed using microtubules (data not shown) and axonemal microtubules (shown). See Figures S3A and S3B for quantification of off-rates.

In principle, dynein could be converted to a microtubule-attached state by arresting its ATPase cycle at a stage corresponding to strong microtubule affinity (e.g., with ADP or no nucleotide bound). Previous ATPase results obtained with brain-purified dynein in the presence of Lis1 have varied (McKenney et al., 2010; Mesngon et al., 2006; Yamada et al., 2008), perhaps due to heterogeneity between and within dynein preparations. To test for Lis1-induced catalytic arrest in our system, we measured the ATPase activity of dynein with different concentrations of Lis1 and microtubules. The basal ATPase rate of GST-dynein_331 kDa_ alone was activated by microtubules to a maximal rate of 10.8 motor-domain^−1^ s^−1^ (Figure 3A). Addition of Lis1 also activated the basal ATPase rate (Figure 3B), indicating that Lis1 binding alters turnover in one or more of dynein's catalytic AAA+ modules. Interestingly, in the presence of microtubules, the maximal ATPase rate of GST-dynein_331 kDa_/Lis1 retained ∼90% of the value for GST-dynein_331 kDa_ alone (Figure 3B). This was the case even in extremely high Lis1 concentrations (2 μM; data not shown). Thus, in the presence of microtubules, dynein/Lis1 can continue to hydrolyze ATP, indicating that Lis1-mediated motility regulation is more complex than a simple arrest of the ATPase cycle.

**Figure S3:**
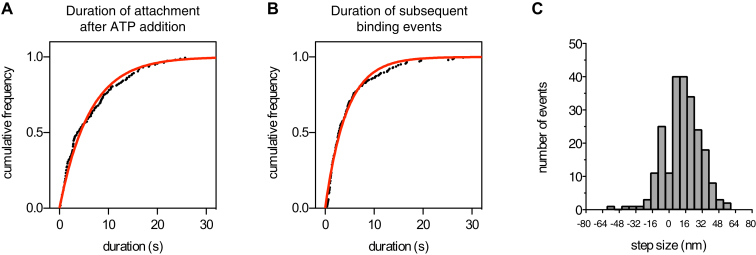
Further Characterization of Dynein Behavior in the Presence of Lis1, Related to Figures 3 and 4 (A and B) In the single-molecule microtubule release assay (Figures 3C–3F), perfusion of ATP into the system caused monomeric dynein_331 kDa_ molecules to rapidly dissociate from the microtubule (typically within 1 s of ATP addition). In the presence of Lis1, dynein molecules remain attached for extended periods. (A) A cumulative frequency plot of the duration of dynein_331 kDa_-microtubule attachments after the addition of ATP. Data points (black) are fit by a one-phase exponential decay (red), with a decay constant of 0.162 ± 0.002 s^−1^ (± error of the fit). The *R*^2^ value is 0.9855. (B) A cumulative frequency plot of the durations of subsequent rebinding events of dynein_331 kDa_ to the microtubule in the presence of ATP and Lis1. The data are fit by a one-phase exponential decay with a decay constant of 0.230 ± 0.002 s^−1^. The *R*^2^ value is 0.9887. (C) In high-precision Qdot stepping experiments, step sizes of GST-dynein_331 kDa_ in the presence of 800 nM Lis1 and 1 mM ATP could be measured. The 1D step size distribution is similar to that previously reported for GST-dynein_331 kDa_ alone in rate-limiting (10 μM) ATP conditions (Reck-Peterson et al., 2006). The probability of back stepping is also the same (0.2) in the presence or absence of Lis1. The histogram includes 223 steps from 10 moving dynein molecules.

In a canonical mechanochemical cycle, dynein's motor domain dissociates from the microtubule after ATP binding. In a dynein dimer, this allows one motor to detach and undergo a forward excursion along the microtubule while the partnering motor remains bound. To test whether Lis1 alters this mechanochemical coupling, we developed an assay to visualize the ATP-sensitive interactions of individual motor domains with microtubules (Figure 3C). Monomeric, fluorescently labeled dynein_331 kDa_ molecules were bound to surface-immobilized microtubules in the absence of nucleotide, inducing strong “rigor” binding. The molecules appeared as stationary, diffraction-limited spots by total internal reflection fluorescence (TIRF) microscopy and as vertical lines in kymographs (Figure 3D). Addition of ATP caused monomeric dynein_331 kDa_ molecules to rapidly dissociate from the microtubule (Figure 3E, arrow). Dissociation was not due to laminar flow or dilution, as the molecules remained attached after perfusion with buffer (Figure 3D, arrow). After ATP-induced dissociation, monomeric dynein_331 kDa_ was free to diffuse away from the microtubule. Subsequent rebinding events were short and probably correspond to single turnovers of ATP (Figure 3E). The presence of Lis1 caused two main differences in these behaviors. First, instead of dissociating rapidly after ATP addition, dynein_331 kDa_ remained bound to the microtubule for extended periods (average duration of ∼6 s) before detaching (Figures 3F and S3A). Second, the duration of rebinding events was similarly prolonged (Figure S3B). Thus, Lis1 slows the microtubule off-rate of dynein in ATP conditions. Multiple ATP hydrolysis cycles are likely to occur during these extended periods of microtubule attachment (see Figure 3B and data below). These results suggest a model in which Lis1 alters communication between dynein's catalytic AAA+ modules and microtubule-binding stalk, allowing the motor domain to remain tightly bound to the microtubule even in high ATP concentrations.

### Impact of Lis1 on Dynein Stepping Behavior

**Figure 4:**
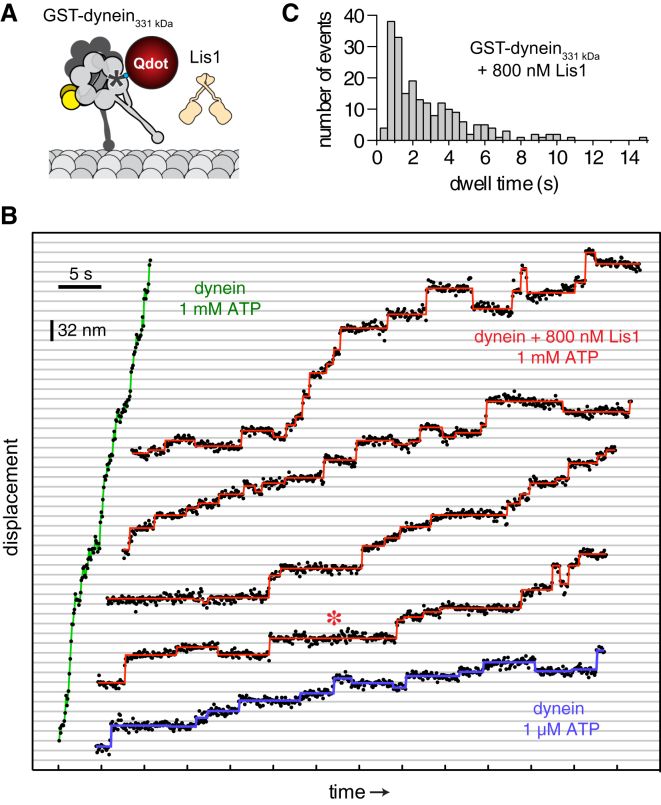
Stepping Behavior of Dynein/Lis1 (A) Diagram of Lis1 and GST-dynein_331 kDa_ labeled at its C terminus with a Qdot. (B) Examples of GST-dynein_331 kDa_ high-precision stepping traces in the presence of 1 μM ATP (blue), 1 mM ATP (green), or 1 mM ATP and 800 nM Lis1 (red). Raw data are shown as black circles. Steps detected by an automated step-finding algorithm (see Extended Experimental Procedures) are depicted with colored lines. The red asterisk highlights an example of a long pause (dwell) by dynein/Lis1. Data were acquired every 100 ms. See also Figure S3C. (C) Histogram showing dwell times between steps by GST-dynein_331 kDa_ in the presence of Lis1, acquired from 209 steps from 10 moving dynein molecules.

A prediction of this model is that Lis1 will cause the motor domains in a dynein dimer to remain attached to the microtubule for longer periods between steps, giving rise to slower motion. If this prediction is correct, then it might be possible to observe individual steps even at a physiological ATP concentration (∼1 mM) without hindering load, conditions under which dynein's steps have previously been too rapid to detect. To test this, we attached a bright, photostable quantum dot (Qdot) to the C terminus of one of dynein's motor domains (Figure 4A; Reck-Peterson et al., 2006). Dynein's movement along microtubules could then be tracked with nanometer precision and a temporal resolution of 100 ms.

As expected, free GST-dynein_331 kDa_ moved rapidly in 1 mM ATP, and high-precision traces showed smooth rather than stepwise motion, consistent with a stepping rate that matches or exceeds the temporal resolution (Figure 4B, green trace). In contrast, movement in the presence of Lis1 was slow and exhibited distinct steps, with pauses (“dwells”) intervening between abrupt forward and occasional backward displacements (Figure 4B, red traces). This Lis1-induced stepping behavior is reminiscent of free dynein in 1 μM ATP, in which the stepping rate is limited by the rate of ATP binding (Figure 4B, blue trace). The dynein step size distribution in the presence of 1 mM ATP and Lis1 (Figure S3C) was similar to that previously reported for dynein alone at low ATP concentration (Reck-Peterson et al., 2006). Occasional larger displacements in the Lis1 traces may correspond to “bursts” of multiple rapid steps at the rate observed for free dynein in 1 mM ATP (Figure 4B, green trace) and could reflect Lis1 dissociation events. In summary, we conclude that the slow movement of dynein/Lis1 is not due to smaller forward steps or more frequent back steps (Figure S3C); rather, it is accounted for by longer dwells between steps.

Most frequently, dynein/Lis1 paused for ∼1 s between steps (Figure 4C), and longer dwells lasting up to 15 s can be seen in the traces (Figure 4B, asterisk). For comparison, the average duration of an ATP turnover in steady-state assays was ∼0.1 s (Figure 3B). The dwells we observe between individual steps in the presence of Lis1 may be related to the pauses previously seen between longer movements in mammalian dynein (see Figure S3 of McKenney et al., 2010). During Lis1-induced pauses, the displacement fluctuations of the Qdot were equivalent to those seen for dynein alone while tightly bound to the microtubule waiting for ATP to bind (in each case, SD = ±4 nm). Together, these results show that Lis1 causes dynein to remain tightly attached to the microtubule for typically more than a second in high concentrations of ATP. This is a pronounced change from dynein's canonical stepping pattern.

### Lis1 Binds Between Dynein's Microtubule-Binding and ATPase Domains at AAA3/4

**Figure 5:**
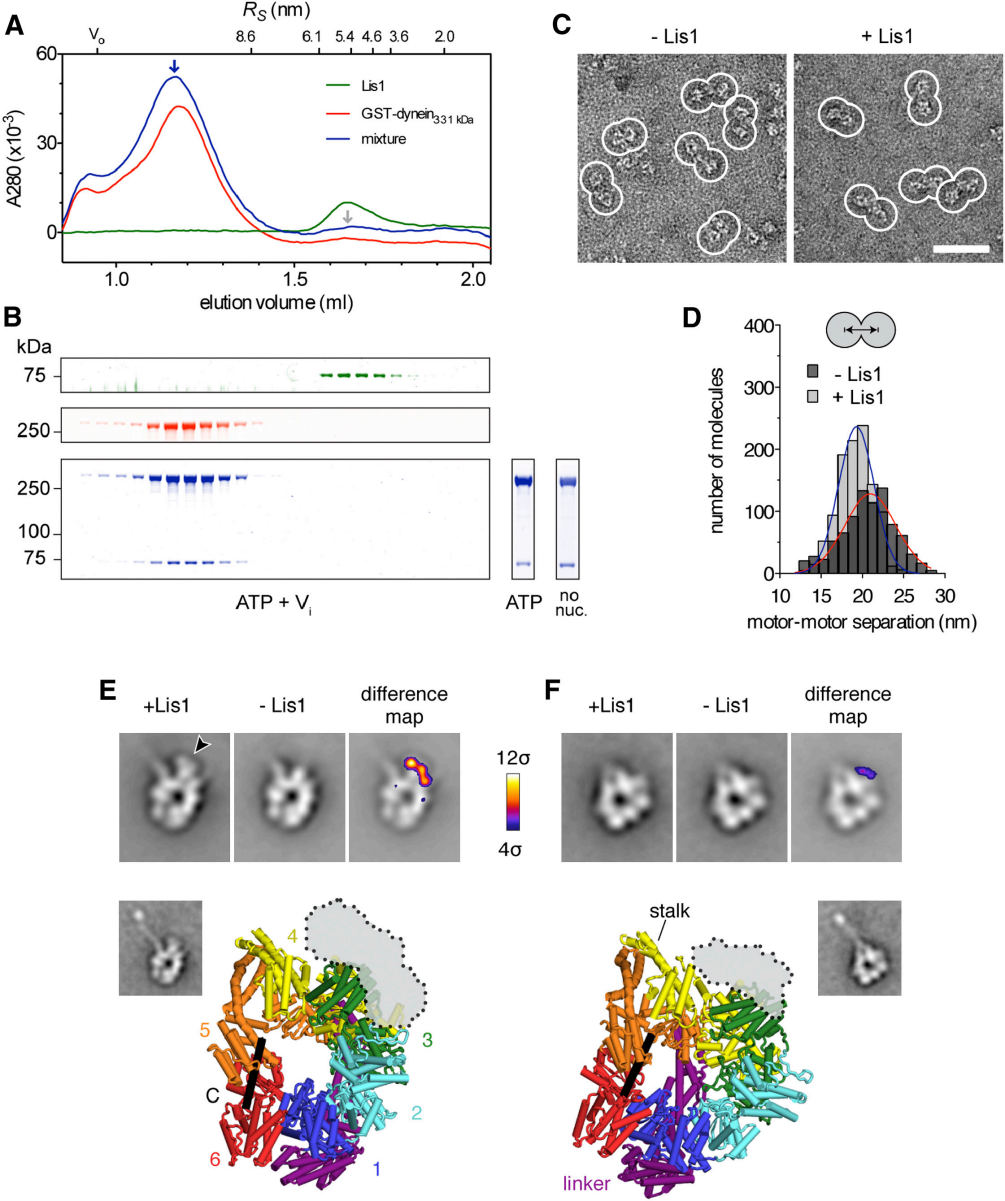
Purification and Structure of the Dynein/Lis1 Complex (A) Purification of the dynein/Lis1 complex by size-exclusion chromatography. Traces show the elution profiles of Lis1 (green), GST-dynein_331 kDa_ (red), and a mixture of both proteins (blue) in ATP + V_i_ buffer conditions. Complex formation is indicated by the coelution of dynein and Lis1 (blue arrow) and depletion of free Lis1 (gray arrow). Elution volumes of standards with known Stokes radii (*R*_*S*_) and the void volume (V_0_) are shown above. (B) SDS-PAGE of size-exclusion chromatography fractions, colored as in (A). GST-dynein_331 kDa_ and Lis1 coelute in a complex (blue bands) in ATP + V_i_ buffer conditions (main panel) as well as in ATP and no-nucleotide conditions (lower right: peak fractions). (C) Negative-stain EM images of GST-dynein_331 kDa_ alone (− Lis1) and bound to Lis1 (+ Lis1). Examples of paired motor domains are outlined in white. The scale bar represents 50 nm. (D) Histogram showing motor-motor separation distances for dynein dimers alone (dark gray) or bound to Lis1 (light gray). Each distribution is fit with a Gaussian (*R*^2^ values of 0.9443 and 0.9830, respectively). Motor-motor separation of dynein alone is 21.0 ± 0.2 nm (± error of the fit) and the SD is 3.1 ± 0.2 nm (n = 854). In the presence of Lis1, the motor-motor separation is 19.3 ± 0.1 nm and the SD is 2.1 ± 0.1 nm (n = 1067). The motor-motor separation is significantly reduced in the presence of Lis1 (p < 0.0001, Welch t test), and the variation in motor-motor spacing is significantly smaller (p < 0.0001, f test). (E and F) Analysis of the Lis1 binding site on dynein. The two main views of the dynein motor domain following single-particle analysis are shown (top view [E] and right view [F]). In each case, the upper row shows an average of the dynein/Lis1 complex (left panel), dynein alone (middle panel), and the difference map between these images overlaid on the dynein average (right panel). Differences are shown at 4σ above the mean and colored according to the chart. Prominent extra density in the dynein/Lis1 complex is indicated (arrowhead in E). The window width corresponds to 26.4 nm. Lower: the difference peak overlaid on the corresponding view of the yeast dynein motor domain crystal structure (PDB 4AKI; Schmidt et al., 2012), as determined by projection matching (see Figures S4B and S4C). The stalk, linker, AAA+ modules (1–6), and C-terminal region (C) are indicated. Insets show class averages revealing the full length of the stalk and microtubule-binding domain at its tip, which are truncated in the crystal structure.

We next explored the structural basis for the striking effects of Lis1 on dynein's mechanochemistry. Using size-exclusion chromatography, we were able to purify the complex of GST-dynein_331 kDa_ and Lis1 in buffer supplemented with ATP + V_i_, ATP, or no added nucleotide (Figures 5A and 5B). Complex formation was evident from the coelution of dynein and Lis1 (Figures 5A, blue arrow, and 5B) and depletion of free Lis1 (Figure 5A, gray arrow). This differs from a previous report in which interactions were detected in the presence of ATP + V_i_ (thought to trap the prepowerstroke state of dynein) but not in nucleotide-free conditions (McKenney et al., 2010). It may be that Lis1 affinity in the no-nucleotide state is stronger with dimeric, yeast dynein compared with the monomeric, mammalian dynein construct used in earlier work. We used electron microscopy (EM) to investigate structural differences between the free dynein and dynein/Lis1 fractions in the absence of nucleotide, allowing direct comparison with the yeast motor domain crystal structure (Schmidt et al., 2012) obtained in the same state.

**Figure S4:**
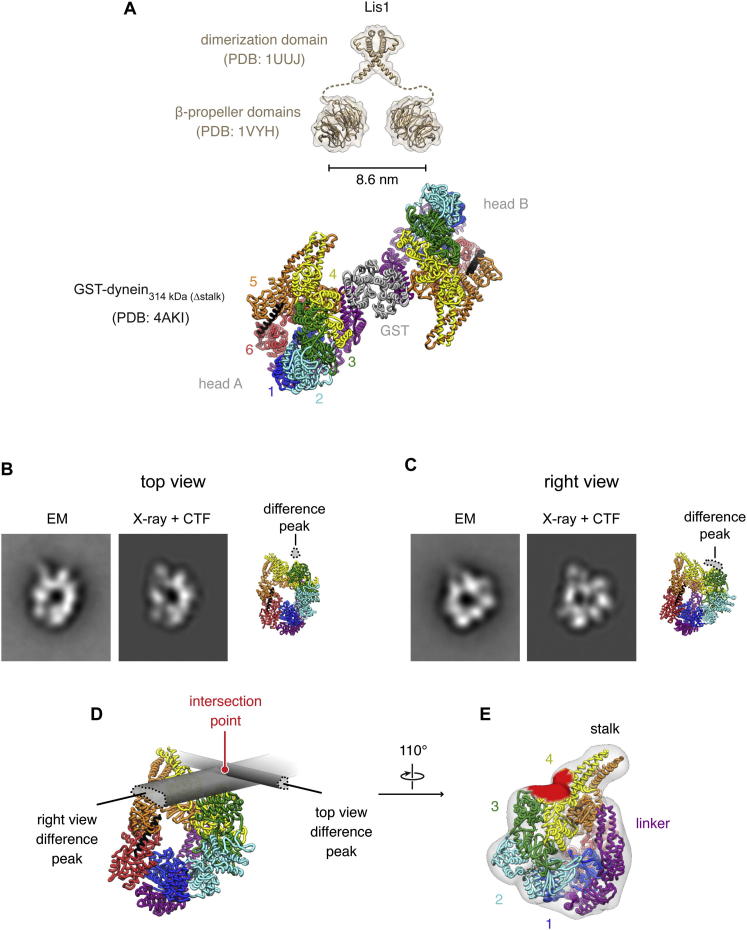
Comparison of EM Data with Dynein and Lis1 Crystal Structures, Related to Figure 5 (A) Crystal structures of Lis1's dimerization domain (PDB 1UUJ; Kim et al., 2004) and β-propeller domains (PDB 1VYH; Tarricone et al., 2004) shown to scale with respect to the crystallized GST-dynein_314 kDa (Δstalk)_ dimer (PDB 4AKI; Schmidt et al., 2012). The 17 amino acids unresolved in the Lis1 crystal structures are represented with dashed lines. Lis1's dimerization domain is shown in the putative “open scissor” conformation (Kim et al., 2004; Tarricone et al., 2004). The two dynein heads, dimerized by GST, are indicated. Lis1's β-propeller domains are in principle capable of spanning the two dynein motor domains in this construct. Whether this can occur when dynein is microtubule-bound is unknown. (B and C) Cross correlation was used to determine orientations of the yeast dynein motor domain X-ray structure (PDB 4AKI; Schmidt et al., 2012) corresponding to the two main views of the motor seen by EM. The X-ray structure was Fourier transformed, treated with a contrast transfer function to simulate the imaging conditions in the EM experiment, back-transformed, and projected at evenly spaced angular intervals (3° between projections). The projections were then aligned to the EM averages (left panels). For each view, the best scoring projection (middle panels) and a corresponding cartoon representation of the X-ray structure (right panels) are shown. Based on the analysis in Figure 5, the difference peaks corresponding to the additional density in the presence of Lis1 are overlaid in each view (dotted outlines). (D) When the Lis1 difference peaks from the two views are combined (effectively bringing the right panels in [B] and [C] into mutual register), they are consistent (i.e., they intersect in three dimensions). The intersection point indicates the approximate 3D position of Lis1 with respect to dynein suggested by this analysis. (E) View of the dynein motor domain, with regions within 25 Å of the intersection point colored in red (25 Å is the radius of the Lis1 β-propeller domain). This putative Lis1 binding surface lies principally on the edge of the ring at AAA4, toward the linker face, and encompasses amino acids within AAA4 whose mutation impairs Lis1 binding (see Figures S5A–S5C). In X-ray structure PDB 4AKI, the truncated N-terminal end of the linker lies close to the putative Lis1 binding surface but does not directly overlap. In constructs with the full-length linker (as seen in *Dictyostelium* dynein structure PDB 3VKG; Kon et al., 2012), direct linker-Lis1 contact may be possible.

Figure 5C shows negative-stain EM images of GST-dynein_331 kDa_ both alone and bound to Lis1. Molecules from the two samples have the same general form, with the paired motor domains adopting a range of relative orientations about the GST moiety. However, the two motor domains are slightly closer and less variably spaced in the presence of Lis1 (Figure 5D), suggesting that Lis1 might bind between them (see Figure S4A).

We next mapped the Lis1 binding site on dynein using single-particle image processing. Images of individual motor domains were extracted from the dimer micrographs, aligned, classified into similar groups, and averaged, revealing a range of molecular views in the data sets. The two main views of the motor domain are close to those previously seen in other dyneins (Figures 5E and 5F; Burgess et al., 2003; Roberts et al., 2009). Detail was sufficient to match these views unambiguously to orientations of the yeast dynein crystal structure (Figures S4B and S4C). Both the coiled-coil stalk and the microtubule-binding domain at its tip are also resolved in class averages (Figures 5E and 5F, insets), further aiding interpretation of the images.

In the presence of Lis1, there is a pronounced extra density on the margin of dynein's motor domain, near the emergence of the stalk (Figure 5E, arrowhead). This observation is quantified in a difference map, where the additional density gives rise to a strong difference peak (Figure 5E, right panel). Analysis of the second main view (Figure 5F) yields a difference peak in the same position. This extra Lis1 density in both views overlaps with AAA3/4 (Figures 5E and 5F, lower; Figures S4B–S4E). We detect no significant density changes near AAA1 in the presence of Lis1, contrary to earlier reports that Lis1 binds AAA1 (Sasaki et al., 2000; Tai et al., 2002).

**Figure 6:**
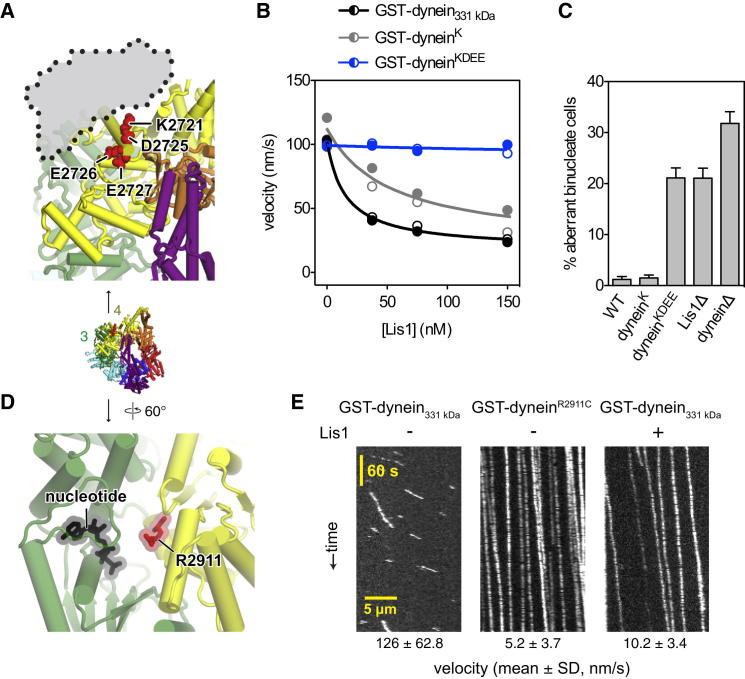
Mutational Analysis of the Lis1 Binding Site and Intersubunit Communication at AAA3/4 (A) Close-up view of the putative Lis1/dynein interface at AAA3 (green) and AAA4 (yellow). The Lis1 difference peak is shown with a dotted outline, overlaid on the yeast dynein crystal structure (PDB 4AKH; Schmidt et al., 2012). Red spheres depict four highly conserved amino acids (K2721, D2725, E2726, and E2727) chosen for mutagenesis. See also Figure S5A. The entire dynein motor domain is shown in the inset below for reference. (B) Mutation of a single amino acid (K2721E; GST-dynein^K^) in the first helix of AAA4 in dynein impairs Lis1-mediated velocity reduction, which is virtually abolished by a quadruple mutation (K2721A, D2725G, E2726S, and E2727G; GST-dynein^KDEE^). Data for GST-dynein_331 kDa_ and GST-dynein^K^ were fit as described in Figure 1G. For GST-dynein_331 kDa_: *R*^2^ = 0.9962; *V*_0_ = 102.2 ± 1.681 nm/s, *F*_max_ = 0.825 ± 0.028, and *K*_1/2_ = 15.68 ± 2.85 nM. For GST-dynein^K^: *R*^2^ = 0.9406; *V*_0_ = 112.6 ± 6.155 nm/s, *F*_max_ = 0.825 ± 0.068, and *K*_1/2_ = 52.18 ± 16.78 nM. Data points from two independent experiments are shown (open and closed circles; n > 203 per data point; see also Table S2C). (C) Quadruple mutation in the first helix of AAA4 causes an aberrant binucleate phenotype highly similar to loss of Lis1 (Lis1Δ). The mean and SE of proportion are shown (n > 405 per data point). WT dynein (WT) and dynein with mutation(s) K2721E (dynein^K^) or K2721A, D2725G, E2726S, and E2727G (dynein^KDEE^) are indicated. (D) Close-up view of the AAA4 arginine finger (R2911; red sticks) that reaches into the nucleotide-binding pocket of AAA3. Nucleotide (AMP-PNP) bound in AAA3 is shown in black stick representation (PDB 4AKH; Schmidt et al., 2012). (E) Kymographs of single-molecule motility of TMR-labeled GST-dynein^R2911C^ on microtubules. The mean velocity for each condition is shown below (n > 162). GST-dynein_331 kDa_ motility in the presence of buffer (−) or 600 nM Lis1 (+) is shown for comparison. See Figure S5D for in vivo characterization of the R2911C mutation.

**Figure S5:**
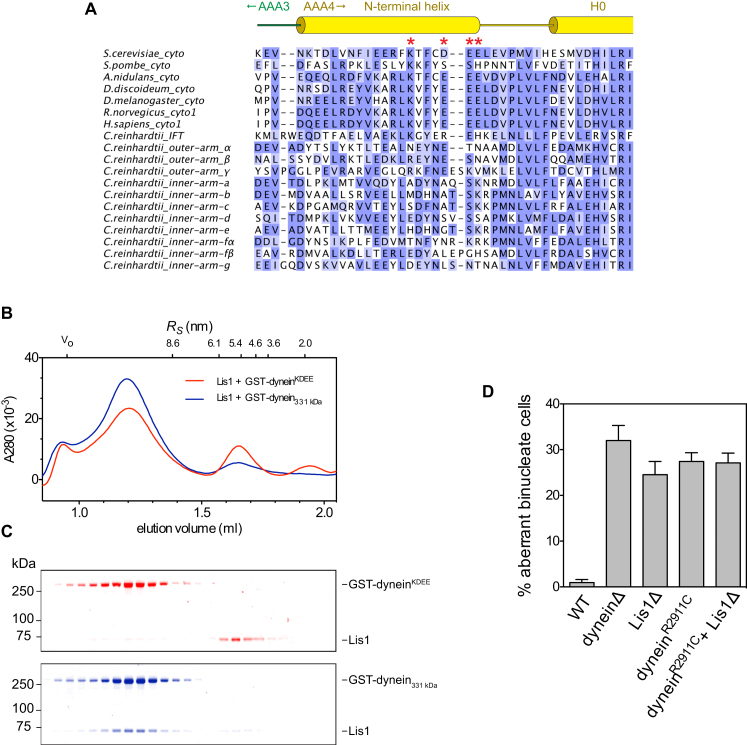
Sequence Analysis and Characterization of Dynein Mutants, Related to Figure 6 (A) Alignment of dynein heavy chain sequences, showing the amino acid conservation at the AAA3/4 junction. The source organism and dynein isoform are indicated for each sequence (cyto, cytoplasmic dynein; IFT, intraflagellar transport dynein; outer/inner-arm, axonemal dynein). Sequences are colored according to BLOSUM62 score. Cylinders show the location of α helices, after PDB 4AKI (Schmidt et al., 2012). The four amino acids implicated in the Lis1 interaction by mutagenesis (red asterisks) are highly conserved among cytoplasmic dynein sequences. A notable exception is *S. pombe*, which also appears to lack a Lis1 ortholog. (B) Four mutations (K2721A, D2725G, E2726S, and E2727G) at the AAA3/4 junction in dynein (GST-dynein^KDEE^) virtually abolish Lis1 binding under conditions in which the intact proteins coelute in a complex by size-exclusion chromatography. Traces show the elution profiles of Lis1 and GST-dynein_331 kDa_ (blue) and Lis1 and GST-dynein^KDEE^ (red) in no nucleotide conditions. Elution volumes of standards with known Stokes radii (*R*_*S*_) and the void volume (V_0_) are shown above. (C) SDS-PAGE of size-exclusion chromatography fractions, colored as in (B). GST-dynein_331 kDa_ and Lis1 coelute in a complex (blue bands), whereas GST-dynein^KDEE^ and Lis1 elute in separate fractions (red bands). (D) In contrast to the case of *A. nidulans*, introduction of the AAA4 arginine finger mutation (R2911C) into *S. cerevisiae* dynein does not rescue the nuclear segregation defect caused by the absence of Lis1 (Lis1Δ). This likely is because Lis1 is essential for proper dynein localization at the microtubule plus end in *S. cerevisiae* (Lee et al., 2003; Sheeman et al., 2003), whereas in *A. nidulans*, this can occur independently of Lis1 (Zhang et al., 2003). The mean and SE of proportion are shown (n > 202 per data point).

To determine whether Lis1 binding at AAA3/4 is responsible for regulating dynein motility, we mutated a series of highly conserved, charged amino acids in dynein at this putative interface (Figures 6A and S5A). Whereas these mutations had little or no effect on dynein's intrinsic motility, Lis1-induced velocity reduction was impaired by a single point mutation (K2721E) and nearly abolished by a quadruple substitution (K2721A, D2725G, E2726S, and E2727G) near the AAA3/4 junction (Figure 6B). These four mutations also virtually abolished Lis1 binding in a gel filtration experiment (Figures S5B and S5C). Furthermore, in living yeast cells, the quadruple substitution produced a nuclear segregation phenotype indistinguishable from that caused by Lis1 deletion (Figure 6C). Thus, we conclude that Lis1 engages dynein at AAA3/4, near the interface between its catalytic ring and microtubule-binding stalk.

### Mutation of Dynein's AAA4 “Arginine Finger” Mimics the Lis1 Effect at the Single-Molecule Level

A pre-existing clue to the involvement of AAA3/4 in Lis1's mechanism comes from genetic studies in *Aspergillus nidulans*. In this filamentous fungus, a single Arg→Cys mutation within AAA4 can partially suppress the phenotype associated with deletion or mutation of the Lis1 gene (*nudF*) (Zhuang et al., 2007). On close inspection, this residue is a strong candidate to form the “arginine finger” motif of AAA4. By analogy to related ring-shaped AAA+ ATPases, this motif is expected to reach from AAA4 into the adjacent nucleotide-binding pocket of AAA3 and help transmit structural changes around the ring (Figure 6D; Erzberger and Berger, 2006; Kon et al., 2012; Schmidt et al., 2012). To investigate the role of AAA4's arginine finger and the consequences of the suppressor mutation, we engineered the equivalent mutation (R2911C) in *S. cerevisiae* dynein. In single-molecule assays, GST-dynein^R2911C^ retained the ability to bind to and move along microtubules. However, its mean velocity was slowed to ∼5 nm/s, and its microtubule encounters were extended in time compared with the parental construct with an intact AAA4 arginine finger (Figure 6E). Strikingly, these properties of the mutant closely resemble dynein behavior in saturating Lis1 concentrations (Figure 6E). As expected, the Lis1Δ phenotype in *S. cerevisiae* is not rescued by the arginine finger mutation (Figure S5D), because here Lis1 is also required for proper dynein localization (Lee et al., 2003; Markus et al., 2009; Sheeman et al., 2003). By contrast, in *A. nidulans*, this mutation partially bypasses the need for Lis1, likely because dynein localization is less dependent on Lis1 in these cells (Zhang et al., 2002). In summary, mutating the arginine finger motif of AAA4 confers motile properties similar to those produced by Lis1; moreover, the same mutation can in part circumvent the requirement for Lis1 in *A. nidulans* (Zhuang et al., 2007).

## Discussion

Our single-molecule and ensemble data illuminate the mechanism by which Lis1 alters cytoplasmic dynein's mechanical behavior and assists in its wealth of cellular functions. Based on these findings, models for Lis1's mechanism must take the following observations into account. First, Lis1 converts single dynein molecules to a microtubule-attached state in a dose-dependent fashion, bringing dynein to a virtual standstill at saturating concentrations. Second, a monomeric Lis1 β-propeller domain, acting on a single dynein motor domain, can elicit these effects. Third, Lis1 alters dynein's mechanochemical coupling: with Lis1 bound, dynein's motor domain can remain attached to the microtubule in physiological ATP concentrations, even while undergoing multiple rounds of hydrolysis. This indicates that Lis1 can alter communication between dynein's ATP hydrolyzing ring and its microtubule-binding stalk. Fourth, Lis1 engages dynein close to the interface between these elements, at AAA3/4.

### A “Clutch” Model for Lis1's Mechanism

**Figure 7:**
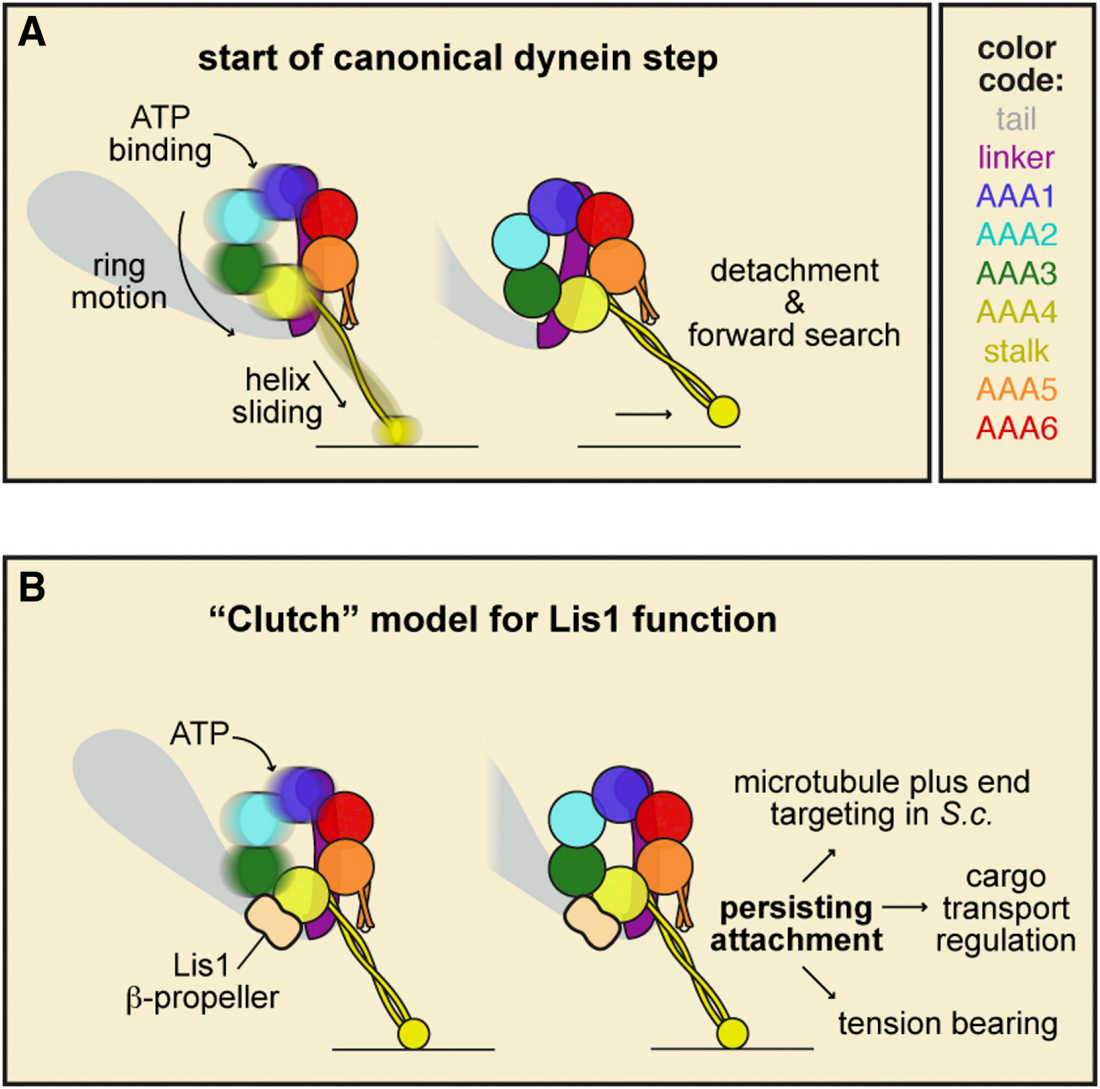
Model for Dynein Motility Regulation by Lis1 (A and B) Model of a canonical dynein step (A) and Lis1-mediated motility regulation (B). See main text for details. For clarity, only one Lis1 β-propeller domain and one dynein heavy chain are shown (both are dimers), and dynein's C-terminal region, which lies on the near face of the ring, is omitted. *S.c*., *S. cerevisiae*.

We bring these observations and previous results together into a model for Lis1's mechanism of action (Figure 7). We first consider a canonical (Lis1-free) dynein stepping cycle that begins with the binding of ATP at dynein's main hydrolytic site (AAA1; Figure 7A, blue). This initiates motions of AAA+ subdomains within the ring, which are translated into sliding between the α helices of the coiled-coil stalk. In turn, the affinity of the microtubule-binding domain at the stalk's tip is weakened; it rapidly detaches from the microtubule and then undergoes a diffusive search for a new binding site.

The main concept of our model is that when Lis1 binds via its β-propeller domain to dynein's ring at AAA3/4, the flow of structural changes from the ring to the stalk is severed (Figure 7B). This function of Lis1 is reminiscent of a “clutch” that alters transmission between dynein's AAA+ “engine” and its track-binding region. Thus, while cycles of ATP binding and hydrolysis can persist within the ring, the microtubule-binding domain at the tip of the stalk retains a strong-binding conformation. As a consequence, the “duty ratio” of the motor (i.e., the fraction of the catalytic cycle spent tightly attached to the track) approaches unity. From a kinetic standpoint, the microtubule off-rate of the motor domain following ATP binding is dramatically slowed, consistent with enhanced microtubule affinity in the ADP-V_i_ state (McKenney et al., 2010). Although the results here show that the dynein-Lis1 complex can be purified in a variety of nucleotide states, our data do not exclude nucleotide-dependent changes in dynein-Lis1 affinity (McKenney et al., 2010); indeed, we think changes in affinity are likely as dynein's ring structure flexes during the ATPase cycle. Interestingly, aspects of Lis1's effects (e.g., constitutively strong microtubule binding and activated ATPase) have been achieved by locking the helices of the stalk's coiled coil in a defined alignment by crosslinking (Kon et al., 2009). Collectively, these results suggest that Lis1 may affect the mobility of the helices in dynein's coiled-coil stalk using a unique allosteric mechanism involving binding near the stalk's base.

### The Role of AAA3/4 in Lis1 Interactions and Dynein Motility

Our structural data, showing that Lis1 binds dynein at AAA3/4 near the interface between its ATP-hydrolyzing ring and microtubule-binding stalk, provide an explanation for how communication between these elements can be regulated. This is in contrast to an earlier report based on interactions between overexpressed fragments of each protein that suggested that Lis1 binds AAA1 (Sasaki et al., 2000; Tai et al., 2002). Our functional studies also support a role for AAA3/4 being the site of Lis1 interaction; mutation of four charged amino acids in AAA4's N-terminal helix severely impairs Lis1 binding, renders dynein nearly completely insensitive to Lis1 in motility assays, and phenocopies a Lis1 null allele in living cells. Furthermore, these charged amino acids are highly conserved in available cytoplasmic dynein sequences (Figure S5A), suggesting that binding at AAA3/4 is likely to be a common feature of Lis1-dynein interactions. A notable exception to this pattern of conservation is found in *Schizosaccharomyces pombe*, which lacks an obvious Lis1 ortholog. The AAA3/4 binding site also provides a simple explanation for why the Lis1-dynein affinity is sensitive to the “neck” region of dynein's tail (Markus and Lee, 2011; Markus et al., 2009), as these domains are closely apposed (Burgess et al., 2003; Roberts et al., 2012).

Although the ATP-driven domain motions within dynein's ring have not yet been delineated, our data and recent structural studies (Kon et al., 2012; Schmidt et al., 2012) suggest that an “arginine finger” motif, reaching from AAA4 into the catalytic pocket of AAA3, contributes to the normal function of cytoplasmic dynein's ring. Our functional studies also show that mutation of this AAA4 arginine finger mimics aspects of Lis1's impact on dynein motility. Based on comparisons with related ring-shaped AAA+ machines, this motif is expected to be important for the hydrolysis of ATP at AAA3 and/or the transmission of structural changes from AAA3 to AAA4 (Erzberger and Berger, 2006). Consistent with this, the motile properties that we observe in the AAA4 arginine finger mutant closely resemble those of mutants with disrupted ATP binding/hydrolysis motifs in AAA3 (Cho et al., 2008; Kon et al., 2004). An important mechanistic distinction between the AAA4 arginine finger mutant and Lis1 is that the mutation appears to substantially reduce ATPase turnover (Zhuang et al., 2007), whereas Lis1 does not. A possible role of ATP hydrolysis in the dynein-Lis1 complex is to keep the system dynamic and therefore adaptable (see below). Because AAA1 is the principal ATPase site in dynein's ring, AAA3 and AAA4 have previously been referred to as “regulatory” AAA+ modules. Here we show that not only do AAA3 and AAA4 regulate the motions within dynein's ring, but they also form the binding site for Lis1, a ubiquitous cytoplasmic dynein regulator.

### The Functional Role of Nudel

We found that Nudel allowed Lis1 to act on dynein at lower concentrations in our single-molecule experiments. This supports a tethering role for Nudel, in which it attaches to dynein's tail (via the intermediate chain of dynein) with one end of its coiled coil and binds to Lis1 with the other (Feng et al., 2000; McKenney et al., 2010; Niethammer et al., 2000; Sasaki et al., 2000; Tarricone et al., 2004; Wang and Zheng, 2011; Żylkiewicz et al., 2011), thus increasing Lis1's effective concentration. However, evidence suggests that within Nudel's tethering role exist additional regulatory layers. For example, an intriguing property of purified mammalian NudE/Nudel proteins is to suppress Lis1's effects on unloaded dynein (Torisawa et al., 2011; Yamada et al., 2008) while still enabling Lis1 to prolong dynein's microtubule attachments under force (McKenney et al., 2010). One possibility here is that Nudel positions Lis1 such that it engages AAA3/4 when dynein is strained. This would allow dynein/Lis1/Nudel to move rapidly until a high load is encountered, at which point Lis1 would engage and resist microtubule detachment. This differs from our results from *S. cerevisiae*, in which Nudel enhanced Lis1's effects on dynein even in unloaded conditions. Therefore, the load-dependent behavior of dynein, Lis1, and Nudel may vary among species or as a function of posttranslational modification.

### Biological Implications

Lis1 has been proposed to facilitate two general steps in the cytoplasmic dynein pathway: preparing dynein for transport and allowing dynein to move large, high-load cargos. Our model (Figure 7) provides a plausible mechanism for both types of Lis1 function and also identifies additional possible roles for dynein/Lis1 in living cells. A common theme in these functions is that by acting as a molecular clutch, Lis1 prolongs dynein's attachments to microtubules.

An initial step in many dynein-mediated transport events is the targeting of dynein to the plus ends of microtubules, which grow and shrink near the cell periphery (Kardon and Vale, 2009). Here, dynein is thought to be loaded with cargo before transporting these cargo molecules toward the microtubule minus end. In cells, Lis1 is also concentrated at microtubule plus ends and can be targeted there either in complex with dynein (as in *S. cerevisiae* and possibly mammalian cells) or separately (as in *A. nidulans* and *Ustilago maydis*; Lee et al., 2003; Lenz et al., 2006; Sheeman et al., 2003; Yamada et al., 2008; Zhang et al., 2002). In either scenario, our data indicate that binding of Lis1 to dynein at the microtubule plus end will bias dynein toward a microtubule-attached state. This could increase dynein's residence time at the microtubule plus end, assist in the kinetics of cargo loading, and/or form the starting configuration for motility toward the minus end. By binding near the neck region of dynein's tail, Lis1 may also be involved in “unmasking” the tail to promote interactions with cargo (Egan et al., 2012; Lenz et al., 2006; Markus and Lee, 2011; Markus et al., 2009).

In *S. cerevisiae*, the cue for transferring dynein onto its cargo protein could also involve the controlled assembly of dynein with the dynactin complex (Markus and Lee, 2011; Markus et al., 2009; Woodruff et al., 2009). Formation of the dynein/dynactin complex may help displace Lis1 and Nudel (McKenney et al., 2011) and convert dynein from a strongly microtubule-attached state (with Lis1 bound) to a rapidly moving processive state (with dynactin bound).

A second, more recently proposed role of Lis1 is to adapt dynein for tasks involving high loads, such as transporting and exerting tension on large organelles and cellular structures (McKenney et al., 2010). By binding near the base of dynein's microtubule-binding stalk and causing it to resist detachment, Lis1 appears ideally suited to facilitate dynein's tension-bearing roles. It remains to be seen whether the ATP-driven movements of dynein's mechanical element (the “linker”) persist with Lis1 bound (Burgess et al., 2003; Kon et al., 2005; Roberts et al., 2009, 2012). If so, this might allow dynein/Lis1 to perform repeated “tugs” on cargo while attached to the microtubule, thereby maintaining tension. Alternatively, Lis1 might regulate the linker domain's motions, as the distal end of the linker lies in close proximity to the Lis1 binding site at AAA3/4 (Figure S4E). The impressive duration of dynein's microtubule attachments in the presence of Lis1 also provides clues to additional possible functions in vivo. A subset of macromolecules and organelles, including mRNAs and the Golgi apparatus, appears to require dynein for retention at specific sites in the cytoplasm (Delanoue and Davis, 2005; Ding et al., 2009; Lam et al., 2010). An idea compatible with our data is that tension-bearing dynein/Lis1 complexes are responsible for anchoring these cargos in place.

An important question is how Lis1 might assist in the rapid, dynein-driven transport of large organelles, as was recently reported in neuronal cells (Pandey and Smith, 2011; Yi et al., 2011). Given that the velocity of these movements (∼300 nm/s) exceeds those expected from dynein in saturating Lis1 concentrations (Yamada et al., 2008), one possibility is that Lis1 is substoichiometrically associated with dynein on such cargos. Alternatives include Lis1 allowing the summation of multiple individual dynein forces (McKenney et al., 2010), and Nudel modulating the dynein-Lis1 interaction in a load-sensitive manner. Quantifying the endogenous copy number of dynein, Lis1, and Nudel on these cargos by live-cell imaging would help distinguish among these possibilities.

In the other classes of cytoskeletal motors, different motile properties have been achieved by gene duplication and divergent evolution. In the case of cytoplasmic dynein, our results reveal that Lis1 can generate mechanical diversity by regulating the structural changes that propagate through dynein's large motor domain.

## Experimental Procedures

### Protein Expression, Purification, and Labeling

The *S. cerevisiae* strains used in this study are listed in Table S1. Dynein constructs were purified and labeled essentially as described previously (Reck-Peterson et al., 2006). For modifications and details of the Lis1 and Nudel purifications, see Extended Experimental Procedures. Protein concentrations were determined by comparisons with standards using Bradford protein assays or SDS-PAGE with SYPRO Red (Invitrogen) staining. All protein concentrations (dynein, Lis1, Nudel, and α/β-tubulin) are expressed for the dimer, with the exception of Lis1_ΔN_, for which the monomer concentration is given.

### Single-Molecule Microscopy and ATPase Assays

Assays are described in detail in the Extended Experimental Procedures. Briefly, motility assays were assembled using flow chambers, and fluorescently labeled dynein molecules and microtubules were visualized by TIRF microscopy (Qiu et al., 2012; Reck-Peterson et al., 2006). Microtubule-activated ATPase assays were performed using the EnzChek phosphatase kit (Molecular Probes; Cho et al., 2008; Reck-Peterson et al., 2006).

### Size-Exclusion Chromatography and EM

GST-dynein_331 kDa_ (350–400 nM), Lis1 (700–800 nM), or a mixture of both proteins was preincubated for 10 min at 4°C under the indicated nucleotide conditions (200 μM Mg-ATP + 200 μM sodium vanadate, 200 μM Mg-ATP alone, or no added nucleotide). Samples were fractionated on a Superose 6 PC 3.2/30 column equilibrated with buffer and nucleotide as required. For negative-stain EM, peak fractions were stained with 1% uranyl formate and imaged using a Tecnai G2 Spirit microscope, as detailed in the Extended Experimental Procedures.

Extended Experimental Procedures **Yeast Strain Construction**

The *S. cerevisiae* strains used in this study are listed in Table S1. The endogenous genomic copies of *DYN1*, *PAC1*, *NDL1*, and *NIP100* (encoding the dynein heavy chain, Lis1, Nudel, and dynactin subunit p150^Glued^ orthologs, respectively) were deleted or modified using PCR-based methods as previously described (Longtine et al., 1998). Transformations were performed using the standard lithium acetate method (Guthrie and Fink, 1991) and screened by colony PCR. Point mutants were generated using either the PCR stitching method or QuikChange site-directed mutagenesis (Stratagene) and verified by DNA sequencing.

**Nuclear Segregation Assays**

Log-phase cells growing at 30°C were transferred to 14°C for 16 hr. Cells were fixed with 75% ethanol and visualized using a Nikon 80i upright microscope equipped with a Hamamatsu C8484-03 monochrome camera and controlled by Metamorph software. The percentage of aberrant binucleate cells was calculated as the number of binucleate cells divided by the sum of dividing wild-type and binucleate cells.

**Protein Expression, Purification, and Labeling**

Cultures of *S. cerevisiae* for protein purification were grown, harvested, and frozen as previously described (Reck-Peterson et al., 2006). Dynein constructs were purified via their ZZ tag, labeled with HaloTag-TMR or HaloTag-PEG-biotin ligands (Promega), and eluted into a modified TEV buffer (50 mM Tris-HCl [pH 8.0], 150 mM potassium acetate, 2 mM magnesium acetate, 1 mM EGTA, 10% glycerol, 1 mM DTT, 1 mM PMSF, and 0.1 mM Mg-ATP). Nudel was similarly purified via its ZZ tag, with the exception that the lysis buffer additionally contained a phosphatase inhibitor cocktail (Roche), and all buffers lacked Mg-ATP.

Lis1 constructs were purified via His_8_ and ZZ tags. Lysed cells were resuspended in Buffer A (final concentrations: 50 mM potassium phosphate [pH 8.0], 150 mM potassium acetate, 150 mM NaCl, 2 mM magnesium acetate, 5 mM β-mercaptoethanol, 10% glycerol, 0.2% Triton X-100, 0.5 mM Pefabloc, and 1 mM PMSF) supplemented with 10 mM imidazole (pH 7.5). Subsequent steps were at 4°C unless indicated. The lysate was clarified by centrifugation at 264,900 *g* for 1 hr. The supernatant was incubated with Ni-NTA agarose (QIAGEN) for 1 hr, transferred into a column, washed three times with Buffer A + 20 mM imidazole, and eluted with Buffer A + 250 mM imidazole. Eluted protein was then incubated with IgG sepharose beads (Amersham Pharmacia) for 1 hr, transferred into a column, and washed twice with Buffer A + 20 mM imidazole and once with TEV buffer (10 mM Tris-HCl [pH 8.0], 150 mM KCl, 10% glycerol, 0.2% Triton X-100, 1 mM PMSF, and 1 mM DTT). Lis1 was released from beads via incubation with TEV protease for 1 hr at 16°C, resulting in cleavage from the His_8_-ZZ tag.

**Single-Molecule Microscopy and Analysis**

Fluorescently labeled molecules were visualized using an Olympus IX-81 TIRF microscope with a 100X 1.45 N.A. oil immersion TIRF objective (Olympus) and four CW diode-pumped solid-state lasers (405 nm and 640 nm cubic lasers [Coherent], and 491 nm and 561 nm lasers [Cobolt]). Laser power at the objective was 1.5–4 mW. Images were recorded with a 100 ms exposure on a back-thinned electron multiplier CCD camera (Hamamatsu) controlled by Metamorph software.

Single-molecule motility assays were performed using flow chambers as previously described (Case et al., 1997). For standard single-molecule dynein motility assays, dynein was labeled with TMR, and microtubules contained ∼10% biotin-tubulin for surface attachment and ∼10% Atto647-, HyLite488-, or fluorescein-tubulin (Cytoskeleton) for visualization. The imaging buffer consisted of 30 mM HEPES (pH 7.2), 50 mM potassium acetate, 2 mM magnesium acetate, 1 mM EGTA, 10% glycerol, 1 mM DTT, 20 μM taxol, 1.25 mg/ml casein, 1 mM Mg-ATP, and an oxygen scavenger system. Images were recorded every 2 s for 10 min, and velocities, durations of microtubule association, and run lengths were calculated from kymographs generated in ImageJ (National Institutes of Health). Runs > 3 s or > 0.5 μm were measured in duration or run length analysis, respectively. All curve fitting was performed in Prism5 (GraphPad). For microtubule gliding assays, GFP-tagged dynein constructs were attached to the coverglass using a polyclonal anti-GFP antibody, and microtubules contained 10% TMR-tubulin. For high-precision stepping experiments, dynein-PEG-biotin molecules were labeled with 655 Qdot streptavidin (Invitrogen) under conditions that yield monovalent Qdot attachment (Reck-Peterson et al., 2006). Images were recorded every 100 ms, and fluorescent spots were fitted with a 2D Gaussian function to precisely localize their position. Steps were detected from the displacement records using a custom MATLAB (The MathWorks) program (Qiu et al., 2012).

For single-molecule microtubule release assays, we used modified flow chambers (Tanner et al., 2009) with inlet and outlet tubes such that buffer conditions could be changed during imaging via a syringe. Monomeric, TMR-labeled dynein_331 kDa_ molecules were bound to immobilized microtubules or axonemal microtubules in the absence of Mg-ATP for 2 min, and then washed with low-salt imaging buffer (lacking potassium acetate) supplemented with 800 nM Lis1 where indicated. After 10 s of imaging (frame rate of 100 ms), the flow chamber was perfused with buffer supplemented with 5 mM ATP and 800 nM Lis1 as indicated.

**Crosslinking Assay**

Glutaraldehyde crosslinking was used to probe the dimerization status of intact Lis1 and Lis1_ΔN_. Proteins (0.5 μM) in crosslinking buffer (30 mM HEPES [pH 7.2], 50 mM potassium acetate) were incubated ± 0.05% glutaraldehyde for 5 min at ambient temperature. Reactions were quenched by the addition of 25 mM Tris-HCl (pH 8.0). As a negative control, proteins were pretreated with 4% LDS to denature them.

**ATPase Assays**

ATPase assays were performed using an EnzChek phosphatase kit (Molecular Probes) as previously described (Cho et al., 2008; Reck-Peterson et al., 2006). The final reaction consisted of 20 nM GST-dynein_331 kDa_, 0 or 140 nM Lis1, 0-10 μM taxol-stabilized microtubules, 2 mM Mg-ATP, 200 μM 2-amino-6-mercapto-7-methyl-purine riboside, 1 U/ml purine nucleoside phosphorylase, and assay buffer (30 mM HEPES [pH 7.2], 50 mM potassium acetate, 2 mM magnesium acetate, 1 mM EGTA, 1 mM DTT, and 10 μM taxol). A SpectraMax384 plate reader (Molecular Devices) was used to monitor the coupled reaction at OD360 every 5 s for 5 min. Data were fit according to Nishiura et al. (2004).

**Size-Exclusion Chromatography**

For size-exclusion chromatography, GST-dynein_331 kDa_ (350–400 nM), Lis1 (700–800 nM), or a mixture of both proteins was preincubated for 10 min at 4°C under the indicated nucleotide conditions (200 μM Mg-ATP + 200 μM sodium vanadate, 200 μM Mg-ATP alone, or no added nucleotide). Samples were fractionated on a Superose 6 PC 3.2/30 column using an ÄKTAmicro system (GE Healthcare) that had been equilibrated with gel filtration buffer (50 mM Tris-HCl [pH 8.0], 150 mM potassium acetate, 2 mM magnesium acetate, 1 mM EGTA, 5% glycerol, and 1 mM DTT) with nucleotide as required. Fractions (50 μl) were analyzed by SDS-PAGE on 4%–12% Tris-Bis gels (Invitrogen) with SYPRO Red staining (Invitrogen), and imaged using an ImageQuant 300 gel imaging system (BioRad). Peak fractions were pooled and flash frozen in liquid nitrogen. Stokes radii were determined by comparisons with protein standards (thyroglobulin, apoferritin, β-amylase, alcohol dehydrogenase, albumin, and carbonic anhydrase [BioRad]).

**EM and Image Processing**

Peak chromatography fractions containing the GST-dynein_331 kDa_/Lis1 complex or GST-dynein_331 kDa_ alone were diluted to 30 μg/ml in gel filtration buffer lacking DTT and glycerol. Specimen (5 μl) was applied to freshly glow-discharged, carbon-coated EM grids and stained with 1% uranyl formate solution. Micrographs were acquired using a Tecnai G2 Spirit microscope operating at 120 kV with a LaB_6_ electron source and a 4k × 4k CCD camera (Gatan US4000). The nominal magnification was 49,000X, giving a sampling of 2.2 Å/pixel at the object level. Single-particle images were selected from the micrographs using BOXER (Ludtke et al., 1999) and processed using SPIDER (Frank et al., 1996) as described previously (Roberts et al., 2009). For measurement of the motor-motor separation, images of dynein dimers were aligned by reference-free methods and classified into groups with an average of five images per class. The centroid positions of the two motor domains in each class average were recorded, and the separation was calculated using arithmetic commands in SPIDER. For mapping of the Lis1 binding site, individual motor domains were extracted from the dimer images using a soft-edged circular mask. The two data sets (±Lis1) were coaligned based on features within the motor domain and separated by classification into characteristic views. Difference maps were calculated between normalized averages from each data set (mean pixel intensity = 0, SD = 1; equal number of particles in each average). Crystal structures were displayed with the use of PyMOL (v1.5.0.1; Schrödinger) and Chimera (Pettersen et al., 2004) software.

## Supplemental Information

Document S1. Tables S1 and S2

Document S2. Article plus Supplemental Information
