# Supplementary material for: Lis1 Acts as a “Clutch” between the ATPase and Microtubule-Binding Domains of the Dynein Motor
Source: Cell. 2012 Aug 31;150(5):975–86. doi: 10.1016/j.cell.2012.07.022 (PMC3438448; doi:10.1016/j.cell.2012.07.022)
Supplement: Document S1. Tables S1 and S2 [file mmc1.pdf]

Table S1. Yeast Strains

Strain	Genotype	Figure(s)<br>Reference
RPY1	W303a (*MATa his3-11,15 ura3-1 leu2-3,112 ade2-1 trp1-1*)	Figures 1, 6, S5<br>(Eshel et al., 1993)
RPY218	W303a *pep4Δ::HIS5 prb1Δ PAC11-13xMYC-TRP1 ZZ-Tev-GFP-3xHA-DYN1-gs-DHA-*Kan^R	Figure S2<br>(Reck-Peterson et al., 2006)
RPY266	W303a *pep4Δ::HIS5 prb1Δ PAC11-13xMYC-TRP1 ZZ-Tev-GFP-3xHA-DYN1-gs-DHA-*Kan^R *pac1Δ::klURA3*	Figures 2, S2<br>(Reck-Peterson et al., 2006)
RPY708	W303a *pep4Δ::HIS5 prb1Δ PAC11-13xMYC-TRP1 ZZ-Tev-GFP-3xHA-DYN1-gs-DHA-*Kan^R *PAC1-3xFLAG*	Figures 2, S2<br>This work
RPY710	W303a *pep4Δ::HIS5 prb1Δ PAC11-13xMYC-TRP1 ZZ-Tev-GFP-3xHA-DYN1-gs-DHA-*Kan^R $PAC1^{3\text{-}129\Delta}$*-3xFLAG-klURA3*	Figures 2, S2<br>This work
RPY753	W303a *pep4Δ::HIS5 prb1Δ* P_{GAL1}*-ZZ-Tev-GFP-3xHA-GST-*$DYN1_{331\ kDa}$*-gs-DHA pac1Δ::klURA3 ndl1Δ::cgLEU2*	Figures 2-6, S2-S5; Table S2<br>This work
RPY780	W303a *pep4Δ::HIS5 prb1Δ ZZ-Tev-3xHA-DYN1-gs-DHA-*Kan^R *nip100Δ pac1Δ::*$Hygro^R$ *ndl1Δ::cgLEU2*	Figures 1, S1; Table S2<br>This work
RPY806	W303a *ndl1Δ::*Kan^R	Figure 1<br>This work
RPY809	W303a *pac1Δ::*$Hygro^R$	Figures 1, 6, S5<br>This work
RPY831	W303a *PAC1-g-1xFLAG-ga-SNAP-*Kan^R	Figure 1<br>This work
RPY842	W303a *pep4Δ::HIS5 prb1Δ* P_{GAL1}*-*HIS_8*-ZZ-Tev-PAC1-g-1xFLAG-ga-SNAP-*Kan^R *dyn1Δ::cgLEU2 ndl1Δ::*$Hygro^R$	Figures 1-6, S1-S5; Table S2<br>This work
RPY844	W303a *pep4Δ::HIS5 prb1Δ PAC11-13xMYC-TRP1* P_{GAL1}*-ZZ-Tev-GFP-3xHA-*$DYN1_{331\ kDa}$ *pac1Δ::*$Hygro^R$	Figures 2, S3<br>This work
RPY870	W303a *NDL1-ga-SNAP-Tev-ZZ-klTRP1*	Figure 1<br>This work
RPY873	W303a *pep4Δ::HIS5 prb1Δ NDL1-ga-SNAP-Tev-ZZ-klTRP1*	Figures 1, S1<br>This work
RPY1042	W303a *pep4Δ::HIS5 prb1Δ* P_{GAL1}*-8HIS-ZZ-Tev-*$PAC1^{3\text{-}129\Delta}$*-g-1xFLAG-ga-SNAP-*$Kan^R$ *dyn1Δ::cgLEU2 ndl1Δ::*$Hygro^R$	Figures 2, S2<br>This work
RPY1116	W303a *pep4Δ::HIS5 prb1Δ ZZ-Tev-GFP-3xHA-DYN1-gs-DHA-*Kan^R *PAC11-13xMYC-TRP1* P_{GAL1}*- PAC1-3xFLAG*	Figure S2<br>This work
RPY1118	W303a *pep4Δ::HIS5 prb1Δ PAC11-13xMYC-TRP1 ZZ-Tev-GFP-3xHA-DYN1-gs-DHA-*Kan^R P_{GAL1}*-PAC1(aa 3-129Δ)-3xFLAG*	Figures 2, S2<br>This work
RPY1176	W303a *pep4Δ::HIS5 prb1Δ* P_{GAL1}*-ZZ-Tev-GFP-3xHA-GST-*$DYN1^{R2911C}$*-gs-DHA pac1Δ ndl1Δ::cgLEU2*	Figure 6<br>This work

RPY1198	W303a *pep4Δ::HIS5 prb1Δ PAC11-13xMYC-TRP1* P_{GAL1}*-ZZ-Tev-GFP-3xHA-DYN1*$_{331\ kDa}$*-gs-DHA-Kan*^{R} *pac1Δ::Hygro*^{R}	Figure 3<br>This work
RPY1199	W303a *dyn1Δ::cgLEU2*	Figures 1, 6, S5<br>This work
RPY1203	W303a *DYN1*^{R2911C}	Figure S5<br>This work
RPY1207	W303a *DYN1*^{R2911C} *pac1Δ::Kan*^{R}	Figure S5<br>This work
RPY1211	W303a *pep4Δ::HIS5 prb1Δ* P_{GAL1}*-ZZ-Tev-GFP-3xHA-GST-DYN1*^{K2721E}*-gs-DHA pac1Δ ndl1Δ::cgLEU2*	Figure 6; Table S2<br>This work
RPY1223	W303a *DYN1*^{K2721E}	Figure 6<br>This work
RPY1237	W303a *DYN1*$^{K2721A,\ D2725G,\ E2726S,\ E2727G}$	Figure 6<br>This work
RPY1238	W303a *pep4Δ::HIS5 prb1Δ* P_{GAL1}*-ZZ-Tev-GFP-3xHA-GST-DYN1*$^{K2721A,\ D2725G,\ E2726S,\ E2727G}$*-gs-DHA pac1Δ ndl1Δ::cgLEU2*	Figures 6, S5; Table S2<br>This work

All strains were made in the RPY1 background with additional changes noted. *DYN1*, *PAC11*, *PAC1*, *NDL1*, and *NIP100* encode the dynein heavy chain, dynein intermediate chain, Lis1, Nudel, and dynactin subunit p150^{glued} orthologs, respectively. Monomeric Lis1 was constructed by removal of amino acids 3-129. *DHA* and *SNAP* refer to the HaloTag (Promega) and SNAP-tag (NEB), respectively. *TEV* indicates a Tev protease cleavage site. P_{GAL1} denotes the galactose promoter, which was used for inducing strong expression of Lis1 and dynein motor domain constructs. Amino acid spacers are indicated by *g* (glycine), *ga* (glycine-alanine), and *gs* (glycine-serine). Genes encoding proteases Pep4 and Prb1 were deleted as noted.

Table S2. Dynein Velocities, Related to Figures 1, 2, and 6

A. Related to Figure 1G

	[Lis1] (nM)	velocity (nm/s)	SD	SE	N
dynein (set 1)	0	102.4	32.34	1.783	329
	37.5	53.25	23.82	1.866	163
	75	44.72	18.48	1.021	328
	150	36.53	15.40	1.192	167
	300	23.06	8.505	0.6662	163
	600	8.622	7.007	0.4980	198
	1200	5.448	3.013	0.1770	290
dynein (set 2)	0	100.5	37.32	2.871	169
	75	47.75	19.29	1.328	211
	150	39.94	14.84	1.241	143
	300	21.18	8.579	0.7832	120
	600	14.97	7.173	0.7138	101
	1200	6.674	3.536	0.3090	131

B. Related to Figure 2C

	[Lis1] (nM)	velocity (nm/s)	SD	SE	N
GST-dynein$_{331\ kDa}$	0	113.0	58.01	5.409	115
(set 1)	4.6875	96.86	57.82	5.091	129
	9.375	64.40	33.59	3.278	105
	18.75	42.18	16.65	1.288	167
	37.5	35.12	12.44	1.126	122
	75	30.73	11.79	0.7725	233
	150	26.50	8.699	0.8183	113
	300	22.20	7.839	0.7651	105
	600	10.21	3.411	0.3063	124
GST-dynein$_{331\ kDa}$	0	115.9	67.20	14.14	227
(set 2)	4.6875	91.51	54.29	12.42	206
	9.375	86.00	47.36	12.50	217
	18.75	61.32	35.93	10.23	214
	37.5	44.70	19.48	10.13	215

	75	37.84	13.62	10.25	209
	150	27.36	9.353	8.946	204
	300	22.24	8.299	7.719	244
	600	13.15	5.915	5.406	201
GST-dynein$_{331\ kDa}$	0	107.6	60.35	9.825	120
(set 3)	4.6875	101.1	53.82	8.975	127
	9.375	77.87	33.58	6.882	128
	18.75	62.28	30.64	5.463	130
	37.5	41.95	14.65	3.830	120
	75	33.55	13.33	3.142	114
	150	21.26	8.083	2.000	113

C. Related to Figure 6B

	[Lis1] (nM)	velocity (nm/s)	SD	SE	N
GST-dynein$_{331\ kDa}$	0	103.4	60.77	3.638	279
(set 1)	37.5	40.68	18.84	1.270	220
	75	32.03	12.16	0.8073	227
	150	23.64	8.789	0.5980	216
GST-dynein$_{331\ kDa}$	0	101.0	57.79	3.810	230
(set 2)	37.5	43.14	20.37	1.413	208
	75	36.43	15.46	1.057	214
	150	26.04	10.41	0.7021	220
GST-dynein^{K}	0	103.9	43.16	2.798	238
(set 1)	37.5	67.17	24.37	1.557	245
	75	55.11	19.75	1.254	248
	150	31.36	9.999	0.5955	282
GST-dynein^{K}	0	120.9	66.89	4.078	269
(set 2)	37.5	81.62	39.65	2.609	231
	75	61.73	31.73	2.222	204
	150	48.72	19.60	1.346	212
GST-dynein^{KDEE}	0	98.39	55.43	3.881	204
(set 1)	37.5	99.06	47.66	3.250	215
	75	94.91	57.06	3.646	245

	150	99.91	57.73	3.984	210
GST-dynein^{KDEE}	0	99.36	89.76	5.688	249
(set 2)	37.5	100.9	68.02	4.268	254
	75	96.56	83.78	5.489	233
	150	93.00	66.32	4.246	244
